# Behind the scenes: how the EMILIN/Multimerin family shapes the cancer landscape

**DOI:** 10.1111/febs.70306

**Published:** 2025-11-05

**Authors:** Evelina Poletto, Naike Casagrande, Emanuele Di Siena, Greta Carobolante, Lucrezia Camicia, Giorgia Schinello, Samanta Muzzin, Enrica Timis, Paola Spessotto, Maurizio Mongiat

**Affiliations:** ^1^ Department of Research and Diagnosis, Division of Molecular Oncology Centro di Riferimento Oncologico di Aviano (CRO) IRCCS Aviano Italy; ^2^ Department of Molecular Sciences and Nanosystems Ca’ Foscary University Venice Italy; ^3^ Department of Medicine University of Udine Udine Italy

**Keywords:** extracellular matrix, glycoproteins, neoplastic stroma, tumor ecosystem, tumor microenvironment

## Abstract

In recent years, the tumor microenvironment has gained recognition as a key regulator of cancer progression. A central component of the tumor microenvironment, the extracellular matrix, undergoes dynamic remodeling during tumor development and plays a crucial role in disease pathogenesis. This review highlights the EMILIN/Multimerin family as a paradigm of the extracellular matrix's diverse and complex functions in cancer. Owing to their intricate domain architecture, these proteins engage in multifaceted interactions, not only directly modulating tumor cell proliferation and migration but also influencing other elements of the tumor microenvironment such as blood, lymphatic vessels, and immune cells. The functional landscape of these interactions is further complicated by proteolytic processing, which can both disrupt native functions and generate bioactive fragments with novel biological activities. Importantly, some of these fragments are detectable in biological fluids, suggesting their potential as predictive or prognostic biomarkers and contributing to the advancement of personalized therapeutic strategies.

## Graphical abstract

The EMILIN/Multimerin family members regulate key hallmarks of cancer—including apoptosis, angiogenesis, metastasis, and tumor microenvironment remodeling. As indicated, their function in immune evasion, drug resistance, and metabolic reprogramming remains largely unexplored. Here, we provide an overview of the multifaceted roles of these molecules in tumor growth and progression. Created with BioRender.com.

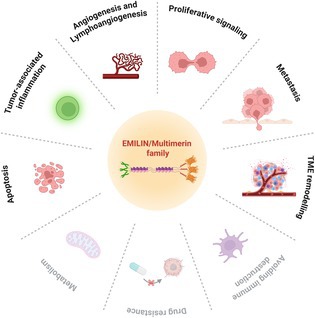

**Abbreviations**
AKT: protein kinase B
BRAF: B‐rapidly accelerated sarcoma
BV: blood vessel
CAF: cancer‐associated fibroblast
CCN: conserved, cysteine‐rich, and N‐terminal domain‐containing protein
ccRCC: clear cell renal carcinoma
CD: cluster of differentiation
CLEC: C‐type lectin domain family member
CLL: chronic lymphocytic leukemia
CNTNAP: contactin‐associated protein family member
CSF: colony‐stimulating factor
CTLA: cytotoxic T‐lymphocyte antigen
CTLD: C‐type lectin domain
CXCR: C‐X‐C chemokine receptor
DISC: death‐inducing signaling complex
DR: death receptor
EC: endothelial cell
ECM: extracellular matrix
EDEN: EMI‐domain endowed
EGF: epidermal growth factor
ERK: extracellular signal‐regulated kinase
EV: extracellular vesicle
FAK: focal adhesion kinase
FREM: FRAS1‐related extracellular matrix
Her: human epidermal growth factor receptor
HIV: human immunodeficiency virus
HPV: human papillomavirus
IGFBP: insulin‐like growth factor binding protein
IL: interleukin
Jak: Janus kinase
LAG: lymphocyte activation gene
LGG: low‐grade gliomas
LN: lymph node
LRP: low‐density lipoprotein receptor‐related protein
LV: lymphatic vessel
MDSC: myeloid‐derived suppressor cell
MMP: metalloproteinase
NET: neutrophil extracellular trap
PD: programmed cell death protein
PDGF‐: platelet‐derived growth factor
PI3K: phosphoinositide 3‐kinase
SARS‐CoV‐2: severe acute respiratory syndrome coronavirus 2
SCC: squamous cell carcinoma
SNX27: Sorting Nexin 27
STAT: signal transducer and activator of transcription
STIC: serous tubal intraepithelial carcinoma
STIL: serous tubal intraepithelial lesion
TGF: transforming growth factor
TIGIT: T‐cell immunoreceptor with Ig and ITIM domains
TLR: toll‐like receptor
TME: tumor microenvironment
TRAIL: TNF‐related apoptosis‐inducing ligand
TSPAN: tetraspanin
UTR: untranslated region
VEGF: vascular endothelial growth factor
VEGFR: vascular endothelial growth factor receptor
YAP: yes‐associated protein

## Introduction

Cancer is one of the leading causes of death worldwide and represents a significant burden on health systems. It is a heterogeneous disease arising from a series of genetic mutations accumulating in tumor cells, leading to uncontrolled cell growth, invasion, and eventually, metastasis.

While genetic alterations are essential for cancer initiation, tumor progression is strongly influenced by the tumor microenvironment (TME) [1]. The TME is a multifaceted network composed of cellular and noncellular components, which, thanks to dynamic interactions, exert profound effects on cancer cells [2]. These components comprise immune cells, endothelial cells (ECs), cancer‐associated fibroblasts (CAFs), and extracellular matrix (ECM), all of which aid tumor progression, impinging on key processes such as the immune response, angiogenesis, and metastasis [3].

One of the TME components that has attracted mounting interest is the ECM [4]. The ECM is a sophisticated, three‐dimensional network composed of proteins, glycoproteins, proteoglycans, and hyaluronan. In normal tissues, it maintains tissue homeostasis, providing structural support and facilitating biochemical signaling, thus regulating cell adhesion, migration, and differentiation [5, 6].

In cancer, however, the ECM undergoes extensive remodeling. Certain components, including collagen, fibronectin, and hyaluronan, are aberrantly overproduced, leading to the formation of a denser and stiffer matrix that supports cancer cell survival, migration, and invasion [6, 7, 8]. Furthermore, the fibrotic tissue arising from excessive ECM deposition can hinder the effectiveness of the therapeutic agents by limiting penetration into the tumor core [9, 10]. On the contrary, the expression of other ECM molecules is reduced or subjected to proteolytic cleavage by enzymes such as metalloproteinases (MMPs) and other proteases [11]. These degradation processes generate bioactive ECM fragments that often acquire novel functions, facilitating tumor progression [6, 12], and creating new routes for cancer cells to colonize distant organs [11, 13]. As a result, these profound modifications can create a permissive environment that fosters tumor development and progression [6, 7, 14, 15].

The ECM does not only directly influence the behavior of cancer cells but also impinges on other TME components, establishing intricate crosstalks that ultimately shape the tumor fate. Indeed, one of the first endogenous anti‐angiogenic molecules to be discovered was the ECM protein endostatin, a C‐terminal fragment of collagen XVIII [16]. Angiogenesis, the formation of new blood vessels (BVs) from pre‐existing vasculature, is essential for tumor growth as it ensures a continuous supply of oxygen and nutrients. For this reason, it has long been viewed as a promising therapy target [17, 18], with anti‐angiogenic approaches now approved for cancer treatment [19, 20]. Besides endostatin, numerous ECM molecules were subsequently demonstrated to regulate angiogenesis [21, 22], adding further complexity to this process. Additionally, the ECM serves as a reservoir of angiogenesis‐active growth factors that are released upon remodeling [23], thereby influencing vascular development in the tumor context.

The ECM also affects lymphangiogenesis [5, 24, 25], the formation of lymphatic vessels (LVs), which plays a key role during the latest stages of tumor progression, facilitating the dissemination of cancer cells to regional lymph nodes (LNs) and distant organs [26, 27]. On the other hand, in concert with the ECM, the lymphatic system also regulates the immune response [28]. While LVs and BVs provide the physical routes for cell trafficking, the ECM influences the immune landscape of the tumors, functioning as a scaffold for immune cell infiltration and modulating their activation through the presentation of growth factors and cytokines [29, 30]. Moreover, the ECM can also contribute to the establishment of an immunosuppressive environment, and its targeting has emerged as a promising strategy to overcome immune evasion and improve immunotherapy efficacy [14]. A deeper understanding of the intricate interplay between the ECM and its remodeling, angiogenesis, lymphangiogenesis, and inflammation is therefore essential to identify new therapeutic strategies and improve patients' survival.

In this review, we will focus on the EMILIN/Multimerin protein family, a group of heterogeneous, widely distributed ECM molecules, characterized by the presence of the cysteine‐rich EMI domain at the N terminus [31].

## The EMILIN/Multimerin family: Protein architecture and localization patterns

**Fig. 1:**
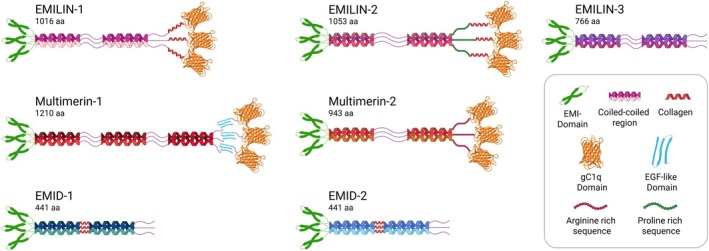
Domain architecture and structural features of the EMILIN/Multimerin (EDEN) protein family. Schematic representation of the structure of the EMILIN/Multimerin (EDEN) family, which comprises seven glycoproteins with an N‐terminal EMI domain (~80 residues). Five members (EMILIN‐1, EMILIN‐2, EMILIN‐3, Multimerin‐1, and Multimerin‐2) feature an EMI domain, α‐helical coiled‐coil region, and (except EMILIN‐3) a C‐terminal gC1q domain. EMID1 and EMID2 share only the presence of the EMI domain and collagen stretches. The EMI domain contains seven conserved cysteines forming three disulfide bonds, facilitating protein interactions and O‐fucosylation. As indicated, many of the members contain unique motifs at the end of the colied‐coil region, prior to the collagen stalk, when present. All members likely form homotrimers, though experimentally confirmed only for EMILIN‐1 and EMILIN‐3. The putative secondary structures of each domain have been represented based on the modeling available at the AlphaFold Protein Structure Database (https://alphafold.ebi.ac.uk/). Created with BioRender.com.

The EMILIN/Multimerin family, also known as EMI domain endowed (EDEN) family [32, 33], consists of seven genes characterized by the presence of the EMI domain (~80 amino acid residues) at the N terminus (Fig. 1). It comprises five large glycoproteins, EMILIN‐1, EMILIN‐2, EMILIN‐3, Multimerin‐1, and Multimerin‐2, where the EMI domain is followed by a long α‐helical coiled‐coil region, and, except for EMILIN‐3, a C‐terminal globular gC1q domain. The two other members, EMID1 and EMID2 (also known as Emu1 and Emu2), contain only the EMI domain and share two collagen stretches and a conserved C‐terminal domain, while they lack structural similarities to other EDEN members [34].

The EMI domain contains seven conserved cysteines forming three disulfide bonds [31], and, in concert with the gC1q domain, it mediates protein–protein interactions and multimerization, and can be O‐fucosylated [35]. In fact, all EDEN family members are predicted to form homotrimers despite this being experimentally proven only for EMILIN‐1 and EMILIN‐3 [36, 37]. However, the role of the gC1q domain in homotrimerization seems to be dispensable, since multimerization occurs also in members lacking this domain, possibly through the EMI domain and collagenic sequences. Both EMILIN‐1 and EMILIN‐2 feature a short collagen‐like Gly‐X‐Y repeat, though EMILIN‐1 forms a weak triple helix while EMILIN‐2 remains less structured due to reduced glycine content. Notably, EMILIN‐1 contains a leucine zipper domain preceding its collagen stalk, contrasting with EMILIN‐2's proline‐rich sequence in the same region. Multimerin‐1 differs from Multimerin‐2 by possessing an RGD cell‐attachment motif and a calcium‐binding EGF‐like repeat, whereas Multimerin‐2 lacks RGD but carries an arginine‐rich sequence. EMILIN‐3 is structurally truncated, terminating after the leucine zipper/collagen region without a gC1q domain. The gC1q domains typically adopt a jelly‐roll β‐sandwich fold, though EMILIN‐1 uniquely has only nine β‐strands with acidic loops that bind integrin α4β1 via a critical glutamic acid motif [32, 38].

Regarding the localization and tissue distribution, EMILINs are classical ECM proteins primarily localized to elastic fiber systems and are expressed during embryonic development [39]. EMILIN‐1 and EMILIN‐2 are found in elastic and oxytalan fibers, particularly in skin, large BVs, and lungs, where they localize at elastin‐fibrillin interfaces [40, 41]. EMILIN‐1 is also crucial in the lymphatic vasculature, with high expression in lymphatic valve leaflets and anchoring filaments [42, 43]. EMILIN‐1 and EMILIN‐2 are highly expressed in the lamina propria adjacent to the gastric islands [44, 45]. EMILIN‐2 is also deposited in the skin [46, 47], and by different cell types, including fibroblasts, astrocytes, and smooth muscle cells; it shows perivascular dermal distribution and increases in scleroderma/fibrosis models [48, 49]. In cardiovascular development, EMILIN‐2 is critical for heart/vessel formation and platelet aggregation [50, 51, 52, 53]. Finally, EMILIN‐2 is also expressed in the cochlea, where it plays a critical function [54, 55, 56].

Originally termed EMILIN‐5, EMILIN‐3 expression was detected at the E8.5–E9.5 stage of embryogenesis in the tail bud region, a structure known to contain a multipotent stem cell population [37]. However, limited data exist on EMILIN‐3 expression and distribution. It is expressed by mesenchymal cells during osteogenesis and in developing limb perichondrium but, unlike other EMILINs, not in the cardiovascular system [37]. Multimerins, instead, exhibit a more cell‐specific distribution. Multimerin‐1 is usually retained in the secretion granules of megakaryocytesand platelets, where it is complexed with coagulation factor V, and ECs [57, 58, 59, 60, 61]. Multimerin‐2, initially identified as EndoGlyx‐1 in a screen for EC surface markers [62], was later cloned and characterized and is specifically expressed by ECs [63]. Its structural similarity to Multimerin‐1 suggests shared ancestry, likely arising from gene duplication [64]. EMID1 and EMID2 are structurally different from the other members of the family, and their expression in adult tissues remains poorly characterized. EMID1 is upregulated during kidney development [34], whereas EMID2 is present in the upper and lower airways [65].

Interestingly, the members of this family are involved in a wide range of biological processes that are central to cancer biology, including tumor cell behavior, angiogenesis, lymphangiogenesis, and the immune response. Importantly, during tumor progression, the function of the EMILIN/Multimerin family can be compromised by the action of proteases, overall promoting malignancy. This review will summarize current knowledge regarding the role of these proteins, emphasizing their profound impact on the TME and tumor progression. We will highlight their emerging potential as molecular targets and/or predictive biomarkers in cancer.

## The EMILIN/Multimerin family: Natural brakes on tumor growth and metastasis

Numerous studies indicate that the ECM generally promotes tumor progression, with its components and corresponding receptors actively favoring cancer cell growth and spreading. Indeed, integrin, which primarily regulates ECM signaling, is a well‐established positive regulator of cell growth [66]. However, only a limited number of ECM proteins, such as decorin [67], perlecan [68, 69], and some members of the CCN family of matricellular proteins [70] are known to exert primarily a tumor‐suppressive function.

**Fig. 2:**
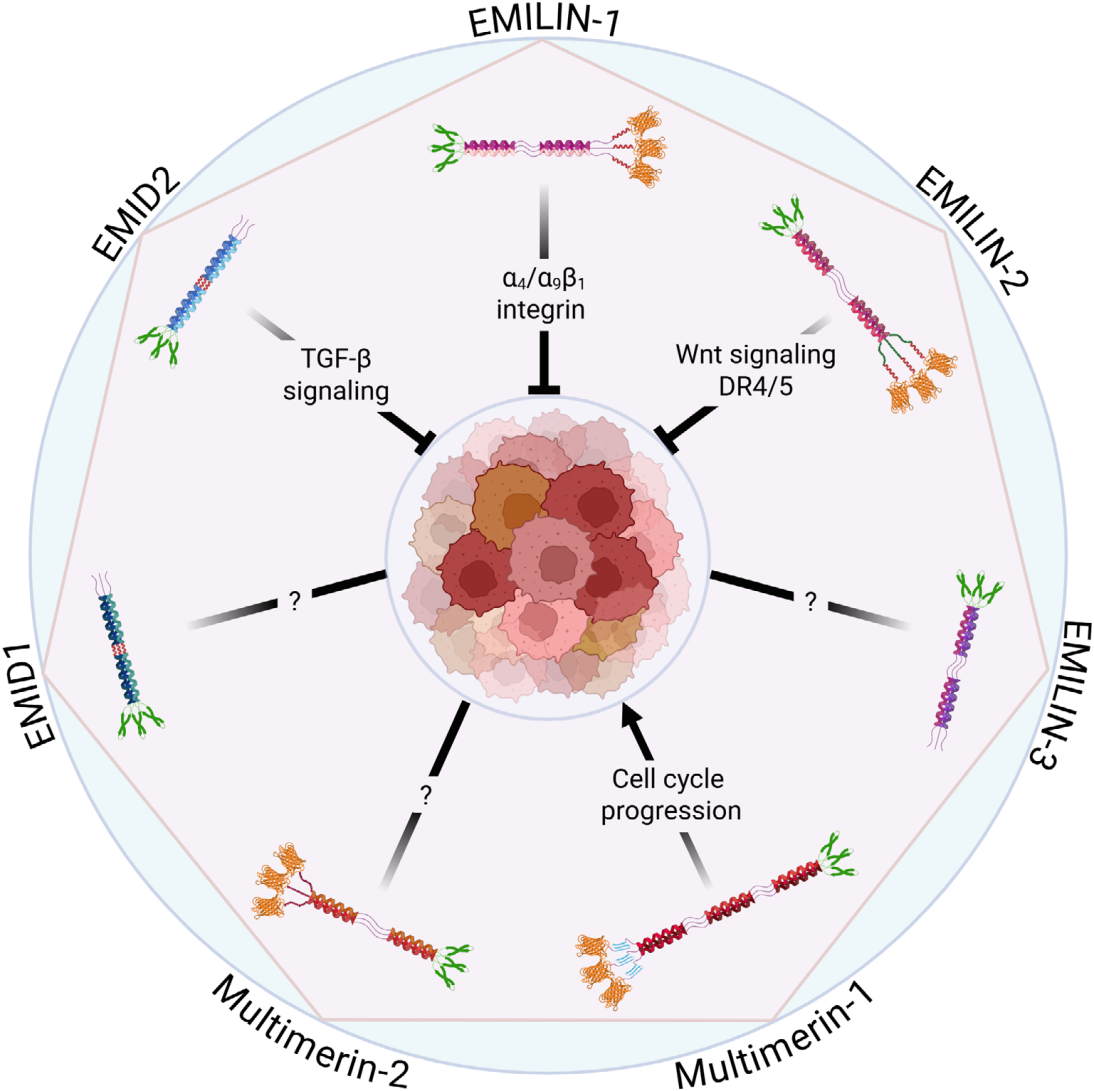
The EMILIN/Multimerin family members modulate cancer cell behavior through receptor‐mediated signaling. The EMILIN/Multimerin family predominantly exerts tumor‐suppressive effects on cancer cells: EMILIN‐1 inhibits proliferation by engaging integrins α4β1 and α9β1, while EMILIN‐2 suppresses Wnt signaling through ligand sequestration and triggers apoptosis via death receptor (DR4/DR5) binding. EMID2 attenuates tumor growth through modulation of TGF‐β signaling. In contrast, Multimerin‐1 uniquely promotes cell cycle progression, though the underlying molecular mechanism remains uncharacterized. The direct effects of EMILIN‐3, Multimerin‐2, and EMID1 on tumor cell behavior remain to be experimentally established. Created with BioRender.com.

The realm of the EMILIN/Multimerin family is peculiar in this perspective since most of the molecules are known to directly or indirectly restrain the growth and progression of tumors. In this section, we will summarize the current knowledge on the role of these molecules in directly affecting cancer cell behavior (Fig. 2).

The glycoprotein EMILIN‐1 was initially identified for its role in maintaining blood pressure through regulation of the TGF‐β signaling pathway [71]. Subsequent studies have broadened its functional profile, linking it to the control of cell proliferation and migration. Collectively, most of the studies have shown that EMILIN‐1 exerts antiproliferative effects, supporting its classification as a tumor suppressor in various cancers. The gC1q domain of EMILIN‐1 engages integrins α4β1 and α9β1, modulating the adhesion and migration of different cell types [43, 72, 73, 74]. Also, the antiproliferative effect of EMILIN‐1 primarily hinges on the engagement of integrins. This was first observed in *Emilin1* ^*−/−*^ mice, which showed epidermal thickening and a higher number of Ki67‐positive cells in both the epidermis and dermis compared to wild‐type counterparts [43]. The underlying mechanism involves PTEN, which mediates the crosstalk between integrins α4β1/α9β1 and the TGF‐β signaling pathway, leading to the downregulation of proliferative cues through ERK inhibition [43]. Similar altered proliferative signals were observed in the context of lymphatic valves [75]. Notably, *Emilin1* ^*−/−*^ mice developed larger lymphangiomas [42], and exhibited accelerated tumor growth with increased tumor size and number in a model of skin carcinogenesis [76]. Similarly, in a HER2‐driven breast cancer model, Δ16HER2/v*Emilin1* ^*−/−*^ mice showed premature tumor onset and increased number of tumor foci [77]. EMILIN‐1's protective role was also evident in colon carcinogenesis, where, through an integrin‐dependent mechanism, its absence led to higher adenoma development [78]. Indeed, in colon cancer cell integrin α4β1 engagement by EMILIN‐1 induces H‐Ras ubiquitination and degradation, suppressing pERK1/2 and proliferation [79]. This mechanism, conserved across different tumor models, reveals EMILIN‐1's unique ability to mediate antiproliferative signaling through α4β1 and α9β1 integrins, a feature distinguishing it from other ligands [80, 81, 82, 83]. Conversely, in chronic lymphocytic leukemia (CLL), the EMILIN‐1–α4β1 interaction promotes prosurvival signals [84], suggesting that EMILIN‐1's effects are context‐dependent and influenced by the TME. In gastric and head and neck squamous cell carcinoma, EMILIN‐1 suppresses cancer cell proliferation and invasion via the TSPAN9 transmembrane protein and the modulation of ERK1/2 and MMP9 [85, 86]. Additionally, in liver cancer, ARID1 promotes EMILIN‐1 expression, thereby inhibiting cell migration and invasion [87]. Similarly, in colorectal cancer, RORB‐driven upregulation of EMILIN‐1 has been found to suppress metastasis [88].

EMILIN‐1 mutations in humans are rare and are associated with connective tissue disorders and neuropathies [89, 90]. In tumors, where matrix remodeling is a hallmark, the loss of EMILIN‐1 function is likely due to proteolytic degradation, rather than gene mutation. In fact, proteases, such as neutrophil elastase, secreted by tumor‐associated inflammatory cells, contribute to its degradation, affecting cancer progression [91, 92, 93, 94, 95]. EMILIN‐1 has been shown to colocalize with neutrophil extracellular traps (NETs), structures made of processed chromatin bound to granular elastase and myeloperoxidase [96], favoring its degradation and impairing its suppressive function [97]. This cleavage occurs within the gC1q domain, which, by contrast, is resistant to the action of other matrix proteases such as MMP14, MMP3, and MMP9, collagenase, and proteinase‐3 [91]. Additionally, EMILIN‐1 fragments have been found in small extracellular vesicles (sEVs) and associate with melanoma and colon cancer progression [98, 99]. Moreover, in breast cancer, elevated levels of EMILIN‐1‐derived peptides were identified in liquid biopsies via LC–ESI–MS/MS spectrometry [100], although the clinical significance of this finding remains elusive.

EMILIN‐1 expression can also be downregulated by promoter hypermethylation, as seen in rhabdomyosarcoma and uterine carcinosarcoma [101, 102]. EMILIN‐1 has emerged as a predictive/prognostic biomarker in several types of cancers. Elevated EMILIN‐1 levels have been correlated with reduced proliferation rates in non‐small‐cell lung cancer [103], and improved therapeutic response in breast cancer [104]. Conversely, elevated EMILIN‐1 levels are observed in osteosarcoma, Ewing's sarcoma, and ovarian carcinoma [105, 106, 107]. The dual role of EMILIN‐1 as both a tumor suppressor and, in some cases, a marker of poor prognosis [108, 109, 110, 111, 112, 113], underscores the complexity of its function, which appears to be highly context‐dependent and influenced by the TME. Further studies are required to validate these contrasting observations and to evaluate the structural and functional integrity of EMILIN‐1 across different tumor settings.

EMILIN‐2 exhibits tumor‐suppressive activity through multiple mechanisms. Studies performed in a sarcoma tumor model demonstrate that it functions as a negative regulator of cancer cell proliferation and can induce apoptosis through the interaction with TRAIL death receptors DR4 and DR5 [114, 115]. Specifically, a 90‐amino acid residues sequence of the N‐terminal coiled‐coil region binds to DR4, leading to formation of the death‐inducing signaling complex (DISC) and subsequent activation of caspase‐8 [116]. Importantly, this apoptotic effect shows selective toxicity toward tumor cells, while leaving normal cells unharmed [116].

In breast cancer, EMILIN‐2 inhibits cell proliferation by suppressing Wnt signaling. The EMI domain of EMILIN‐2 directly binds Wnt1, competes with the Frizzled receptor and reduces LRP6 phosphorylation. This leads to downregulation of β‐catenin, TAZ, and their target genes (e.g., *c‐Myc*, *cyclin D*), decreasing S‐phase entry and phospho‐histone H3‐positive cells [117], overall diminishing breast cancer cell tumorigenicity. Accordingly, ectopic expression of EMILIN‐2 *in vivo* suppressed Wnt signaling, tumor growth, and metastasis. In support of these findings, an *in silico* meta‐analysis of 2878 breast cancer patients revealed an inverse correlation between EMILIN‐2 and Wnt target genes, with high EMILIN‐2 expression associated with improved relapse‐free survival [117]. The EMILIN‐2‐Wnt interaction may also be relevant in HPV‐E6‐driven cervical and other anogenital cancers. Indeed, the oncogenic HPV‐E6 through the interaction with the endosomal protein SNX27, impairs EMILIN‐2 trafficking and deposition, thereby weakening Wnt signaling and favoring cell proliferation [118].

In ovarian cancer EMILIN‐2 emerges as a dual‐function p53 effector that simultaneously regulates proliferation and metabolism [119, 120]. Ovarian cancer arises from serous tubal intraepithelial lesions (STIL) to serous tubal intraepithelial carcinoma (STIC) and eventually established tumor also modulating p53. During tumor progression EMILIN‐2 is detectable in STIL and absent in STIC. Mechanistically, p53 directly activates EMILIN‐2 transcription and its upregulation antagonizes tumor growth and reprograms metabolism by reducing glycolysis and promoting oxidative phosphorylation [119]. These results demonstrate that EMILIN‐2 may inhibit tumor growth engaging different suppressive mechanisms in cancer cells.

In accordance with its tumor‐suppressive function, EMILIN‐2 is frequently downregulated through promoter hypermethylation in multiple cancers. HBV‐related hepatocellular carcinoma exhibits tumor‐specific EMILIN‐2 hypermethylation, compared to adjacent tissue [121]. In breast cancer, it ranks among the top five methylated genes, with methylation correlating with poor prognosis, lymph node metastasis, and hormone receptor‐positive status [122]. Lung adenocarcinoma shows smoking‐associated EMILIN‐2 methylation linked to disease progression and poor overall survival [123]. Also colorectal tumors exhibit EMILIN‐2 methylation in 33% of carcinomas and 32% of adenomas, and this associates with BRAF mutations, lymphatic invasion, and metastasis [124]. In ovarian cancer, EMILIN‐2 is downregulated via promoter hypermethylation, correlating with worse prognosis, while serving as a potential serum biomarker [120, 125]. In melanoma, EMILIN‐2 hypomethylation correlates with better outcomes [126].

The function of EMILIN‐2 in the TME can be also suppressed by degradation even though the responsible enzymes have not been identified. Elevated levels of EMILIN‐2 were detected in the blood serum of patients with esophageal diseases, including high‐grade dysplasia and esophageal adenocarcinoma, compared to disease‐free controls [127]. In gastric carcinomas, EMILIN‐2 is lost across all tumor stages, further suggesting its tumor‐suppressive role [44]. Notably, this suppression occurs independently of disease progression parameters. Intriguingly, when present, EMILIN‐2 expression shows a significant positive correlation with increased microvascular density, as evidenced by CD31 staining patterns [44]. This vascular association suggests potential roles in tumor angiogenesis regulation beyond its direct tumor‐suppressive functions, as detailed in the next section.

Intriguingly, rare EMILIN‐2‐PDGFD fusions in dermatofibrosarcoma protuberans mimic COL1A1‐PDGFB‐driven pathogenesis through PDGFRB activation, whereas, in breast cancer, this fusion associates with aggressive features, including fibrosarcomatous transformation and deep invasion [128, 129, 130].

In contrast with its downregulation in most tumors, EMILIN‐2 is upregulated in cholangiocarcinoma, colon adenocarcinoma, clear cell renal carcinoma (ccRCC), esophageal squamous cell carcinoma (SCC), and low‐grade gliomas (LGGs). Its expression holds a prognostic value in adrenocortical carcinoma, ccRCC, gliomas, testicular germ cell tumors, and uveal melanoma [131, 132, 133]. These contrasting results suggest that the effect of EMILIN‐2 may be context‐dependent. However, it must be taken into account that, while mRNA analysis reveals transcriptional activity, EMILIN‐2 protein levels may not correlate due to extensive post‐transcriptional regulation and ECM remodeling in tumors. This discrepancy necessitates direct protein validation through immunohistochemistry or proteomic analysis to accurately assess EMILIN‐2's functional presence and clinical relevance in these cancer tissues.

The role of EMILIN‐3 in directly modulating cancer cell behavior is poorly characterized. Nonetheless, EMILIN‐3 expression is upregulated in low‐grade gliomas (LGG) where it associates with increased tumor grade and worse prognosis [111, 134], thus functioning as a potential predictive marker in this type of tumor [135, 136]. In colorectal cancer, frequent copy number gains of 20q12 chromosomal regions comprising the *EMILIN‐3* gene were observed [137], and the gene was also found in a signature associated with brain but not liver metastasis [138]. In addition, EMILIN‐3 expression may also affect drug resistance in cancer cells [139]. However, these studies rely solely on database analyses of mRNA expression and provide no information on actual protein levels, processing, or the underlying molecular mechanisms.

Although Multimerin‐1 has been primarily characterized in platelet biology [140], its direct role in cancer has not been thoroughly evaluated. Given the well‐established contribution of platelets to tumor progression [141], Multimerin‐1 may play a significant role in oncogenic processes. Indeed, the calcium‐binding EGF‐like domain of Multimerin‐1 may facilitate protein–protein interactions and regulate cell proliferation, differentiation, and cancer progression [142, 143]. In accordance with its role in tumors, Multimerin‐1 exhibits cancer‐specific expression patterns [144], being downregulated in oropharyngeal squamous cell carcinomas [145], and hepatocellular carcinoma [146], whereas it is upregulated in acute myeloid leukemia [147], pancreatic cancer [148], and cervical cancer [149]. In ovarian cancer, Multimerin‐1 silencing impairs viability, migration, and invasion while increasing apoptosis and disrupting DNA repair pathways [150]. In small‐cell lung cancer, its downregulation associates with an increased sensitivity to cisplatin [151]. Interestingly, Multimerin‐1 fragments have been detected in the body fluids, highlighting its diagnostic potential [149, 152, 153, 154].

As an EC‐specific protein, Multimerin‐2 shows dysregulation in multiple angiogenesis‐related diseases, including myocardial infarction [155], pulmonary hypertension [156], uveitis [157], SARS‐CoV‐2 infection [158], neurodegenerative diseases such as Parkinson [159], and HIV‐associated infections [160]. The potential role of Multimerin‐2 in cancer is underscored by its distinct dysregulation patterns across multiple tumor types, suggesting a possible involvement in tumorigenic processes. In fact, bioinformatic studies show generally reduced Multimerin‐2 mRNA levels in most tumors except certain hematological malignancies and glioblastomas, where its overexpression correlates with poorer survival [111]. In ccRCC, high Multimerin‐2 expression correlates with improved outcomes [161], whereas when mutated in lung adenocarcinoma associates with worse prognosis [162]. However, these gene expression analyses may not be representative of the actual protein levels in the tissues, which are substantially influenced by protease activity, as our published and preliminary data indicate. In fact, the discontinuous Multimerin‐2 deposition in gastrointestinal cancers likely results from MMP2/9‐mediated cleavage [22, 163, 164, 165]. Proteomic analyses reveal a cancer‐specific enrichment of Multimerin‐2 fragments in the body fluids of ovarian [166], lung [167], breast [168], and thyroid cancer patients [169]. Interestingly, recent findings suggest a potential vascular mimicry mechanism in breast cancer through CD93/β1‐integrin/PI3K signaling [170], though clinical validation remains needed. These collective findings position Multimerin‐2 as a multifaceted potential biomarker while highlighting the need to better understand its proteolytic processing and context‐dependent functions across cancer types.

Although the role of EMID proteins in cancer remains poorly understood, EMID1 has emerged as a key regulator in breast and prostate malignancies. Initially identified in highly metastatic murine mammary tumors, EMID1 was upregulated in cells with increased metastatic potential [171]. The knockdown of EMID1 in metastatic breast cancer cells reduced proliferation by delaying cell cycle progression, while promoting cell motility [171]. High EMID1, combined with low Contactin‐Associated Protein Family Member 3 (CNTNAP3) and FRAS1‐Related Extracellular Matrix 1 (FREM1) levels, correlates with improved breast cancer patient survival [172]. However, in lung adenocarcinoma, low EMID1 levels correlate with poorer outcomes, hinting at context‐dependent effects [173]. EMID1 is also upregulated in prostate cancer bone metastases, particularly osteoblastic lesions [174]. Thus, the role of EMID1 in affecting cancer progression and the mechanisms involved remains elusive and requires additional investigations.

Also the role of EMID2 in cancer has not been extensively investigated. Nonetheless, in a recent study, EMID2 was shown to exhibit tumor‐suppressive properties, inhibiting proliferation, migration, and invasion. In fact, EMID2 emerged in a large‐scale screening of secreted proteins as a potent suppressor of tumor dissemination in lung and pancreatic models [175]. Mechanistically, EMID2 blocks TGF‐β maturation, preventing CAF activation and resulting in reduced YAP nuclear localization and decreased ECM stiffness, collectively suppressing invasiveness. EMID2‐overexpressing tumors *in vivo* were smaller and more circumscribed, correlating with improved patient outcomes, thus highlighting its prognostic potential [175]. Whether EMID1 and EMID2 expression is regulated by proteolytic processing remains to be investigated.

## Multifaceted tumor control: How EMILIN/Multimerin proteins orchestrate angiogenic, lymphangiogenic, and immunomodulatory networks in cancer

Among the EMILIN/Multimerin protein family, EMILIN‐1 is the only component known to play a crucial role in the lymphangiogenesis process. In fact, EMILIN‐1 is essential to maintain the integrity of LVs. EMILIN‐1 deficiency results in a significant reduction in anchoring filaments, hyperplasia, enlargement, and in an irregular pattern of superficial and visceral LVs leading to mild lymphedema, impaired lymph drainage and enhanced leakage [42]. These effects are integrin α4β1/α9β1‐dependent in coordination with the VEGFR3, ultimately activating the AKT and ERK pathways [43, 91, 176].

In a lymphedema mouse model, EMILIN‐1 degradation by neutrophil elastase weakens lymphatic EC junctions, worsening lymphedema, which can be reabsorbed using a neutrophil elastase inhibitor, suggesting a possible therapeutic approach [92]. These lymphatic alterations also have implications for cancer metastasis. In syngenic models of melanoma and lung cancer, *Emilin‐1* ^*−/−*^ animals display more LN metastasis compared to *wild‐type* controls [76], likely due to compromised structural integrity of LVs. LVs are key in resolving inflammation [177], accordingly, in gastric cancer, EMILIN‐1 loss associates with chronic inflammation, impaired inflammatory cell clearance, particularly of monocytes and macrophages, and enhanced malignancy [45, 78, 178]. This is likely due to a combination of defective lymphatic clearance and altered expression of inflammatory mediators [45]. EMILIN‐1 loss may indeed impact TGF‐β signaling, influencing also macrophage polarization and immune suppression, thereby further skewing the immune balance toward a chronic inflammatory state [178]. Using single‐cell RNA sequencing and a spatial transcriptomic approach, EMILIN‐1 was found enriched in the tumor area characterized by high cytotoxic CD8+ T‐cell infiltration, in a TGF‐β‐dependent manner [179].

However, in other contexts, high EMILIN‐1 expression has been associated with resistance to radiotherapy [180], potentially due to complex interactions within the ECM and modulation of the immune microenvironment. Further studies are required to better elucidate the crosstalk between EMILIN‐1 and the inflammatory microenvironment.

Beyond its direct effects on cancer cell growth, EMILIN‐2 also significantly influences the TME. In fact, EMILIN‐2‐overexpressing tumors displayed enhanced vascularization [116]. Subsequent studies demonstrated that EMILIN‐2 promotes angiogenesis by binding EGFR on ECs, activating the Jak2‐STAT3 pathway ultimately promoting IL‐8 expression [48]. A similar mechanism also occurs in cancer cells and fibroblasts, where the increased IL‐8 production further drives EC proliferation and migration via CXCR1 [48]. In gastric cancer, EMILIN‐2 expression positively correlates with CD31, used as a BV marker [44], and the increased vascularization of the tumors partly relied on increased expression of IGFBP2, SERPINE1, and VEGFA [44].

The clinical significance of these findings is underscored by observations that tumors with low EMILIN‐2 expression frequently develop dysfunctional vasculature negatively impacting on treatment efficacy [48]. In fact, EMILIN‐2 enhances vascular stability through different mechanisms: (1) It promotes pericyte recruitment through PDGF‐BB and HB‐EGF; (2) it facilitates pericyte adhesion via integrins α5β1 and α6β1; (3) it strengthens EC‐pericyte connection by upregulating N‐cadherin and collagen IV expression [181]. Importantly, the treatment with a pericyte‐recruiting agent improves chemotherapy efficacy by restoring vascular functionality [181]. Particularly noteworthy is the dramatic near‐complete tumor regression achieved in sarcoma mouse models through combination therapy using bevacizumab with EMILIN‐2 fragments, an effect mediated by enhanced vascular normalization and perfusion [116]. Collectively, these findings establish EMILIN‐2 as a promising therapeutic target capable of simultaneously improving vascular stability and enhancing the efficacy of both anti‐angiogenic agents and conventional chemotherapy.

EMILIN‐2 is expressed in the bone marrow [182], and indeed, it has emerged in immune‐related conditions including Kavasaki disease [183], viral infection [158], and age‐related metabolic changes [184]. In cancer, EMILIN‐2‐challenged gastric tumor cells upregulate the expression of several cytokines, including IL‐8, a cytokine involved in both angiogenesis and immune cell recruitment [44]. In ccRCC, high EMILIN‐2 expression correlates with immune checkpoint upregulation (CTLA‐4, PD‐1, LAG3, and TIGIT), enriched T‐cell infiltration, and poor prognosis [131]. In preclinical settings, EMILIN‐2 deficiency correlates with increased PD‐L1 expression and enhanced immunotherapy response, which led to vascular normalization and decreased hypoxia [126]. In CRC, EMILIN‐2 exhibits stage‐dependent immunomodulation: Early lesions from *Emilin‐2* ^*−/−*^ mice show myeloid derived suppressor cells (MDSCs) recruitment via G‐CSF, while advanced tumors display unbalanced M2‐polarized macrophages through a TLR‐4‐dependent mechanism, with low EMILIN‐2 expression predicting poor outcomes [124]. In gliomas, blood–brain barrier disruption enables infiltration of peripheral monocytes/macrophages. In this context, EMILIN‐2 emerged as a discriminator between peripheral macrophages and resident microglia, the brain's predominant macrophage population [185]. Comprehensive *in silico* immune profiling further revealed EMILIN‐2's critical role in shaping immune cell infiltration in LGG [133]. Furthermore, breast cancer spatial analysis revealed EMILIN‐2 as a key prognostic marker, with high expression in tumor (PanCK+) regions predicting poorer survival compared to immune (CD45+) regions [186]. More recently, EMILIN‐2 was discovered as a target for anti‐angiogenic immunotherapy. In mouse melanoma models, EMILIN‐2 vaccination shrank tumors, reduced BVs, and changed immune cell activity [187]. Taken together, these findings position EMILIN‐2 as a key molecule between angiogenesis and immunity, highlighting its potential as both prognostic biomarker and therapeutic target across multiple cancer types.

Studies linking EMILIN‐3 to tumors are limited. However, published evidence suggests that EMILIN‐3 influences the microenvironment with potential contributions during tumor progression. For instance, like other family members, EMILIN‐3 inhibits the maturation of TGF‐β [37], whose role in tumor‐related inflammation is well‐established [188]. Additionally, EMILIN‐3 interacts with heparin through its EMI domain, suggesting a potential role in modulating heparan sulfate proteoglycan‐associated signaling pathways, including those mediated by Wnt, Hedgehog, and BMP ligands [37, 189, 190]. While these functions were characterized in developmental and cutaneous contexts, these findings raise the possibility that EMILIN‐3 may indirectly influence tumor progression through modulation of the immune TME.

While Multimerin‐1 is well‐characterized in platelet biology and hemostasis, its potential influence on the TME remains poorly understood. Multimerin‐1 may play a significant role in angiogenesis, as peptides derived from its EGF‐like domain have been shown to stimulate proliferation in both keratinocytes and ECs [191]. Additionally, Multimerin‐1 may also activate TGF‐β under shear stress, suggesting broader vascular and immune functions [192]. The angiogenic potential, combined with Multimerin‐1's established roles in vascular biology and cell adhesion, positions this molecule as a potential modulator of BV formation in both physiological and pathological contexts. Nonetheless, given the differential expression in various cancers, its involvement in cell adhesion, and modulation of signaling pathways [193, 194], Multimerin‐1 could play a context‐dependent role in shaping the TME, representing an important area for future research.

Multimerin‐2 primarily influences tumor progression by modulating the TME, particularly through the regulation of tumor‐associated vasculature. When ectopically overexpressed in the TME, Multimerin‐2 inhibits tumor growth by impairing vascularization, thereby restricting blood supply [195]. This angiostatic function is mediated by its ability to sequester VEGFA via its glycosylated coiled‐coil domain, thereby suppressing VEGFR2 activation [195, 196]. Mechanistically, Multimerin‐2 reduces phosphorylation of VEGFR2 at residues Y1214 and Y1175, as well as downstream signaling through FAK and p38, ultimately inhibiting EC migration and sprouting angiogenesis [195]. Multimerin‐2 expression is tightly regulated during active angiogenesis. Pro‐angiogenic cytokines suppress Multimerin‐2 mRNA levels, while its proteolytic degradation, primarily by MMP9 and, to a lesser extent, MMP2, further reduces its extracellular deposition [163]. Beyond angiogenesis inhibition, Multimerin‐2 is crucial for maintaining endothelial junction integrity. In Multimerin‐2‐deficient ECs, VE‐cadherin lining becomes discontinuous due to elevated VEGFR2‐Y951 phosphorylation, which promotes VE‐cadherin internalization [197]. Consistent with this, *Multimerin‐2* ^*−/−*^ mice exhibit defective tumor vasculature, increased hypoxia, and impaired drug delivery, reducing therapeutic efficacy [197].

In addition to the indirect VEGFR2 modulation, Multimerin‐2 directly interacts with C‐type lectin domain (CTLD) family receptors, including thrombomodulin, CLEC14A, CD93, and CD248 [198]. These interactions fine‐tune angiogenic processes and influence broader TME dynamics [199, 200, 201]. Buried among other data of a 2012 proteomic screen [202], the Multimerin‐2‐CLEC14A interaction waited several years before its mechanistic importance in angiogenesis was revealed. In two consecutive studies, Multimerin‐2 was demonstrated to bind to CLEC14A via the CTLD domain and to CD93 via the F238 residue, and disruption of this interaction suppresses sprouting angiogenesis and tumor growth [203, 204, 205, 206, 207]. In glioblastoma, Multimerin‐2 colocalizes with CD93‐positive vessels, stabilizing CD93–β1‐integrin interactions and promoting actin cytoskeleton remodeling, EC adhesion, and migration [208, 209]. This complex also guides fibronectin fibrillogenesis, as tumors from *Cd93* ^*−/−*^ mice exhibit reduced vascular fibronectin deposition [208]. Despite its pro‐angiogenic role, CD93, such as Multimerin‐2 [197], also maintains endothelial barrier integrity, limiting cancer cell dissemination [210]. Thus, from the therapeutic point of view, targeting Multimerin‐2‐CD93 with antibodies or nanoparticles may offer novel anti‐angiogenic strategies [211, 212].

Another mechanism by which Multimerin‐2 may affect vascular stability is the interaction with CD248, a receptor expressed by pericytes and stromal cells [204, 213]. Multimerin‐2 enhances pericyte adhesion, and *Multimerin‐2* ^*−/−*^ mice display poor pericyte coverage under both physiological and pathological conditions [197, 214], although a direct role for CD248 in this process remains unconfirmed. Collectively, Multimerin‐2 may act as a molecular bridge between ECs, via CD93/CLEC14A, and pericytes, possibly via CD248, reinforcing vessel stability. However, the complexity of these interactions warrants further investigation.

Emerging evidence also suggests possible crosstalk between Multimerin‐2 and immune‐related pathways. The 5'‐UTR of Multimerin‐2 mRNA is targeted by miR‐1910‐5p via CXCR4 [215], an important chemokine receptor regulating immune cell crosstalk [216]. This finding highlights a potential link between angiogenesis and immune signaling, opening new avenues for research in immune–TME interactions.

Despite the published evidences are scant, EMID1 and EMID2 also seem to shape cancer progression via microenvironmental and immune modulation. EMID1 disrupts cell adhesion, potentially altering stromal interactions and immune infiltration. For instance, in lung adenocarcinoma, high EMID1 correlates with increased B‐cell infiltration [173]. EMID2 impacts on the TME by inhibiting TGF‐β maturation, thus blocking CAF activation and ECM stiffening to reduce invasiveness and normalize the tumor mechanical niche [175]. Further studies are required to better define the role of these elusive molecules in shaping the TME.

## Conclusions

**Fig. 3:**
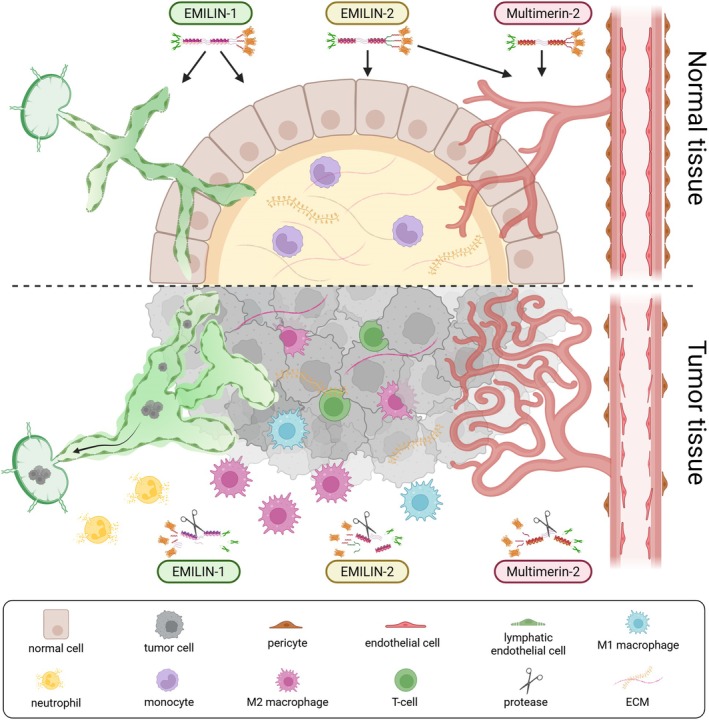
EMILIN/Multimerin proteins regulate the TME homeostasis. In healthy tissues, EMILIN/Multimerin glycoproteins maintain vascular and lymphatic stability while suppressing proliferation through integrin binding (EMILIN‐1/α4β1/α9β1) and Wnt pathway/apoptosis modulation (EMILIN‐2). Tumor‐associated proteases (e.g., MMPs, neutrophil elastase) degrade EMILIN‐1, EMILIN‐2 and Multimerin‐2, with multifaceted consequences in the tumor microenvironment (TME). Degradation of EMILIN‐1 and EMILIN‐2 abolishes their antiproliferative signaling, enabling uncontrolled tumor growth. EMILIN‐1 deficiency further destabilized lymphatic vessels, fostering metastatic dissemination and impairing lymphatic drainage, which sustains chronic inflammation. Conversely, loss of EMILIN‐2 and Multimerin‐2 disrupts vascular integrity, exacerbating hypoxia and compromising drug delivery. Additionally, EMILIN‐2 depletion polarizes macrophages toward a pro‐tumor M2 phenotype, reshaping immunosuppressive networks in the TME. Created with BioRender.com.

Collectively, emerging evidence demonstrate that multiple EMILIN/Multimerin family members play significant roles in tumor biology through both direct and indirect mechanisms (Fig. 3). These glycoproteins show consistently dysregulated expression patterns across various malignancies. However, many of these analyses are based on mRNA levels (Table 1), which do not necessarily correlate with protein levels. Therefore, investigation into the proteolytic processes within the TME that substantially influence protein levels is required to fully understand their role. The structural homology shared among family members raises the intriguing possibility of functional compensation between paralogs, which could explain the complex phenotypes observed in single‐gene perturbation studies.

**Table 1:** Dysregulation of the EMILIN/Multimerin protein family in tumors. CDDP, cisplatin; DFS, disease‐free survival; IF, Immunofluorescence; IHC, Immunohistochemistry; Mass Spec, Mass Spectrometry; N/A, not applicable; OS, overall survival; WB, Western Blot.

Molecule	Cancer type	Method of analyses	Fragment release	Clinical implication	References
EMILIN‐1	Breast cancer	Mass Spec	Serum	Descriptive study	[100]
	Breast cancer	RNA sequencing + spatial transcriptomic	N/A	Correlaion with improved prognosis	[179]
	Breast cancer	cDNA microarray + *In silico* analyses	N/A	correlation with improved therapy response	[104, 180]
	Ewing's sarcoma	*In silico* analyses	N/A	Descriptive study	[106]
	Osteosarcoma	Mass Spec	N/A	Descriptive study	[105]
	Ovarian cancer	mRN/A SAGE aN/Alyses	N/A	Descriptive study	[107]
	Gastric cancer	*In silico* analyses	N/A	Correlation with poor OS	[109]
	Low‐Grade Glioma	*In silico* analyses	N/A	Correlation with poor OS/DFS	[111]
	Oropharyngeal cancer	*In silico* analyses	N/A	Correlation with improved OS	[113]
	Rhabdomyosarcoma	DNA methylation profile	N/A	Descriptive study	[101]
	Uterin carcinosarcoma	DNA methylation profile	N/A	Descriptive study	[102]
	Non‐Small‐Cell Lung cancer	*In silico* analyses + IHC	N/A	Descriptive study	[103]
	Breast cancer	*In silico* analyses	N/A	Correlation with improved OS	[77]
	Gastric cancer	*In silico* analyses + IF	N/A	Descriptive study	[45, 108]
	Head and Neck Squamous Cell carcinoma	Mass Spec + Nanostring	N/A	Descriptive study	[112]
EMILIN‐2	Ovarian cancer	Mass Spec + IHC	N/A	Correlation with improved OS	[119]
	Ovarian cancer	Mass Spec	Serum	Descriptive study	[125]
	Esophageal adenocarcinoma	Mass Spec	Serum	Descriptive study	[127]
	Breast cancer	DNA/RNA sequencing	N/A	Descriptive study	[186]
	Dermatofibrosarcoma	DNA/RNA sequencing	N/A	Descriptive study	[128, 129, 130]
	Renal cancer	*In silico* analyses	N/A	Correlation with poor OS	[131]
	Esophageal adenocarcinoma	Gene expression array	N/A	Descriptive study	[132]
	Low‐Grade Glioma	*In silico* analyses	N/A	Correlation with poor OS	[111, 133]
	Hepatocellular carcinoma	Bisulfite sequencing	N/A	Descriptive study	[121]
	Ovarian cancer	mRNA expression + IHC	N/A	Correlation with poor OS	[120]
	Colorectal cancer	Bisulfite sequencing	N/A	Descriptive study	[124]
	Breast cancer	CpG island microarray	N/A	Correlation with poor OS	[122]
	Breast cancer	*In silico* analyses	N/A	Correlation with poor OS	[117]
	Lung cancer	CpG island microarray	N/A	Correlation with poor OS	[123]
	Melanoma	*In silico* analyses	N/A	Correlation with poor OS and immunotherapy efficacy	[126]
	Gastric cancer	IF	N/A	Descriptive study	[44]
EMILIN‐3	Low‐Grade Glioma	*In silico* analyses	N/A	Correlation with poor OS	[111, 134, 135]
	Colorectal cancer	DNA sequencing	N/A	Descriptive study	[137]
	Metastatic colorectal cancer	Nanostring analyses	N/A	Descriptive study	[138]
	Low‐Grade Glioma	*In silico* analyses	N/A	Correlation with improved OS	[136]
Multimerin‐1	Pediatric Acute myeloid Leukemia	mRNA expression	N/A	Correlation with poor OS and relapses	[147]
	Pancreatic cancer	Mass Spec	Plasma	Descriptive study	[148]
	Cervical cancer	Mass Spec	Urine	Descriptive study	[149]
	Non Small Cell Lung cancer	Mass Spec	Plasma	Correlation with CDDP resistance	[151]
	Ovarian cancer	WBs/ELISA	Saliva	Descriptive study	[152, 153]
	Multiple myeloma	Mass Spec	Serum	Descriptive study	[154]
	Oropharyngeal squamous cell carcinoma	mRN/A microarray	N/A	Descriptive study	[145]
	Hepatocellular carcinoma	Mass Spec	Serum	Descriptive study	[146]
Multimerin‐2	Renal cancer	*In silico* analyses	N/A	Correlation with improved OS	[161]
	Ovarian cancer	Mass Spec	Plasma	Descriptive study	[166]
	Lung adenocarcinoma	Mass Spec	Pleura effusion	Descriptive study	[167]
	Breast cancer	Mass Spec	Urine	Descriptive study	[168]
	Thyroid cancer	Mass Spec	Tumor tissue	Descriptive study	[169]
	Low‐Grade Glioma	*In silico* analyses	N/A	Correlation with poor OS	[111]
	Lung adenocarcinoma	AI application on TCGA	N/A	Correlation with poor OS	[162]
	Gastric Cancer	IF	N/A	Descriptive study	[164, 165]
EMID1	Breast cancer	*In silico* analyses	N/A	Correlation with improved OS	[172]
	Prostate cancer	mRNA array + IHC	N/A	Descriptive study	[174]
	Lung adenocarcinoma	*In silico* analyses	N/A	Correlation with poor OS	[173]
EMID2	Pancreatic cancer	*In silico* analyses	N/A	Correlation with improved OS	[175]
	Lung adenocarcinoma	*In silico* analyses	N/A	Correlation with improved OS	[175]

To substantiate this hypothesis, systematic investigation using conditional knockout mouse models with combinatorial genetic ablation of different family members sharing similar expression patterns would be particularly informative. Such approaches could elucidate compensatory mechanisms while evaluating their collective impact on tumor initiation, growth, and metastatic progression.

While these findings underscore the therapeutic potential of targeting EMILIN/Multimerin proteins, critical knowledge gaps remain. The roles of EMILIN‐3, EMID1, and EMID2 are poorly understood, with the exception of a recent EMID2 study that may catalyze future research. The molecular mechanisms through which EMILIN‐3 and EMID1 modulate the TME are largely unknown, necessitating further investigation. Furthermore, a comprehensive characterization of the expression patterns of this family of molecules across tumor subtypes and stages is needed. Furthermore, the mechanistic basis of their tumor‐modulatory functions requires elucidation, since their multidomain architecture suggests complex interaction with various receptors, cytokines, and signaling pathways. Finally, although their potential as diagnostic and prognostic biomarkers is promising, further validation is essential. Future investigations should focus on quantifying these fragments in biological fluids and rigorously assessing their correlation with clinical outcomes.

Taken together, the pleiotropic nature of EMILIN/Multimerin family members positions them as promising, yet complex, therapeutic targets in oncology, demanding rigorous preclinical validation before considering clinical translation in oncology.

## Conflict of interest

The authors declare no conflict of interest.

## Author contributions

EP, NC, EDS, GC, LC, and GS wrote parts of the manuscript; EDS created the images; SM, ET revised the manuscript; PS and MM conceived the subject, wrote part of the manuscript, and revised it.

## References

[1] Li Z , Li J , Bai X , Huang X & Wang Q (2024) Tumor microenvironment as a complex milieu driving cancer progression: a mini review. Clin Transl Oncol 27, 1943–1952. 39342061 10.1007/s12094-024-03697-w PMC12033186

[2] Hanahan D (2022) Hallmarks of cancer: new dimensions. Cancer Discov 12, 31–46. 35022204 10.1158/2159-8290.CD-21-1059

[3] Quail DF & Joyce JA (2013) Microenvironmental regulation of tumor progression and metastasis. Nat Med 19, 1423–1437. 24202395 10.1038/nm.3394 PMC3954707

[4] Prakash J & Shaked Y (2024) The interplay between extracellular matrix remodeling and cancer therapeutics. Cancer Discov 14, 1375–1388. 39091205 10.1158/2159-8290.CD-24-0002 PMC11294818

[5] Lu P , Weaver VM & Werb Z (2012) The extracellular matrix: a dynamic niche in cancer progression. J Cell Biol 196, 395–406. 22351925 10.1083/jcb.201102147 PMC3283993

[6] Karamanos NK , Theocharis AD , Neill T & Iozzo RV (2019) Matrix modeling and remodeling: a biological interplay regulating tissue homeostasis and diseases. Matrix Biol 75, 1–11. 30130584 10.1016/j.matbio.2018.08.007 PMC6377817

[7] Pickup MW , Mouw JK & Weaver VM (2014) The extracellular matrix modulates the hallmarks of cancer. EMBO Rep 15, 1243–1253. 25381661 10.15252/embr.201439246 PMC4264927

[8] Fejza A , Camicia L , Poletto E , Carobolante G , Mongiat M & Andreuzzi E (2021) ECM remodeling in squamous cell carcinoma of the Aerodigestive tract: pathways for cancer dissemination and emerging biomarkers. Cancer 13, 2759. 10.3390/cancers13112759 PMC8199582 34199373

[9] Minchinton AI & Tannock IF (2006) Drug penetration in solid tumours. Nat Rev Cancer 6, 583–592. 16862189 10.1038/nrc1893

[10] Zhang X , Zhang X , Yong T , Gan L & Yang X (2024) Boosting antitumor efficacy of nanoparticles by modulating tumor mechanical microenvironment. EBioMedicine 105, 105200. 38876044 10.1016/j.ebiom.2024.105200 PMC11225208

[11] Egeblad M & Werb Z (2002) New functions for the matrix metalloproteinases in cancer progression. Nat Rev Cancer 2, 161–174. 11990853 10.1038/nrc745

[12] Hynes RO (2009) The extracellular matrix: not just pretty fibrils. Science 326, 1216–1219. 19965464 10.1126/science.1176009 PMC3536535

[13] Torre‐Cea I , Berlana‐Galán P , Guerra‐Paes E , Cáceres‐Calle D , Carrera‐Aguado I , Marcos‐Zazo L , Sánchez‐Juanes F & Muñoz‐Félix JM (2025) Basement membranes in lung metastasis growth and progression. Matrix Biol 135, 135–152. 39719224 10.1016/j.matbio.2024.12.008

[14] Yuan Z , Li Y , Zhang S , Wang X , Dou H , Yu X , Zhang Z , Yang S & Xiao M (2023) Extracellular matrix remodeling in tumor progression and immune escape: from mechanisms to treatments. Mol Cancer 22, 48. 36906534 10.1186/s12943-023-01744-8 PMC10007858

[15] Walker C , Mojares E & Del Río Hernández A (2018) Role of extracellular matrix in development and cancer progression. Int J Mol Sci 19, 3028. 30287763 10.3390/ijms19103028 PMC6213383

[16] O'Reilly MS , Boehm T , Shing Y , Fukai N , Vasios G , Lane WS , Flynn E , Birkhead JR , Olsen BR & Folkman J (1997) Endostatin: an endogenous inhibitor of angiogenesis and tumor growth. Cell 88, 277–285. 9008168 10.1016/s0092-8674(00)81848-6

[17] Folkman J (1971) Tumor angiogenesis: therapeutic implications. N Engl J Med 285, 1182–1186. 4938153 10.1056/NEJM197111182852108

[18] Ferrara N & Kerbel RS (2005) Angiogenesis as a therapeutic target. Nature 438, 967–974. 16355214 10.1038/nature04483

[19] Ferrara N , Hillan KJ , Gerber HP & Novotny W (2004) Discovery and development of bevacizumab, an anti‐VEGF antibody for treating cancer. Nat Rev Drug Discov 3, 391–400. 15136787 10.1038/nrd1381

[20] Ansari MJ , Bokov D , Markov A , Jalil AT , Shalaby MN , Suksatan W , Chupradit S , Al‐Ghamdi HS , Shomali N , Zamani A *et al*. (2022) Cancer combination therapies by angiogenesis inhibitors; a comprehensive review. Cell Commun Signal 20, 49. 35392964 10.1186/s12964-022-00838-y PMC8991477

[21] Mongiat M , Andreuzzi E , Tarticchio G & Paulitti A (2016) Extracellular matrix, a hard player in angiogenesis. Int J Mol Sci 17, 1822. 27809279 10.3390/ijms17111822 PMC5133823

[22] Andreuzzi E , Capuano A , Poletto E , Pivetta E , Fejza A , Favero A , Doliana R , Cannizzaro R , Spessotto P & Mongiat M (2020) Role of extracellular matrix in gastrointestinal cancer‐associated angiogenesis. Int J Mol Sci 21, E3686. 10.3390/ijms21103686 PMC7279269 32456248

[23] Siddhartha R & Garg M (2023) Interplay between extracellular matrix remodeling and angiogenesis in tumor ecosystem. Mol Cancer Ther 22, 291–305. 36861362 10.1158/1535-7163.MCT-22-0595

[24] Ji R‐C (2006) Lymphatic endothelial cells, lymphangiogenesis, and extracellular matrix. Lymphat Res Biol 4, 83–100. 16808670 10.1089/lrb.2006.4.83

[25] Wiig H , Keskin D & Kalluri R (2010) Interaction between the extracellular matrix and lymphatics: consequences for lymphangiogenesis and lymphatic function. Matrix Biol 29, 645–656. 20727409 10.1016/j.matbio.2010.08.001 PMC3992865

[26] Alitalo K (2011) The lymphatic vasculature in disease. Nat Med 17, 1371–1380. 22064427 10.1038/nm.2545

[27] Montenegro‐Navarro N , García‐Báez C & García‐Caballero M (2023) Molecular and metabolic orchestration of the lymphatic vasculature in physiology and pathology. Nat Commun 14, 8389. 38104163 10.1038/s41467-023-44133-x PMC10725466

[28] Johnson LA (2021) In sickness and in health: the immunological roles of the lymphatic system. Int J Mol Sci 22, 4458. 33923289 10.3390/ijms22094458 PMC8123157

[29] Fejza A , Carobolante G , Poletto E , Camicia L , Schinello G , Di Siena E , Ricci G , Mongiat M & Andreuzzi E (2023) The entanglement of extracellular matrix molecules and immune checkpoint inhibitors in cancer: a systematic review of the literature. Front Immunol 14, 1270981. 37854588 10.3389/fimmu.2023.1270981 PMC10579931

[30] Yang J , Tang S , Saba NF , Shay C & Teng Y (2025) Tumor secretome shapes the immune landscape during cancer progression. J Exp Clin Cancer Res 44, 47. 39930476 10.1186/s13046-025-03302-0 PMC11809007

[31] Doliana R , Bot S , Bonaldo P & Colombatti A (2000) EMI, a novel cysteine‐rich domain of EMILINs and other extracellular proteins, interacts with the gC1q domains and participates in multimerization. FEBS Lett 484, 164–168. 11068053 10.1016/s0014-5793(00)02140-2

[32] Colombatti A , Spessotto P , Doliana R , Mongiat M , Bressan GM & Esposito G (2011) The EMILIN/Multimerin family. Front Immunol 2, 93. 22566882 10.3389/fimmu.2011.00093 PMC3342094

[33] Doliana R , Canton A , Bucciotti F , Mongiat M , Bonaldo P & Colombatti A (2000) Structure, chromosomal localization, and promoter analysis of the human elastin microfibril interfase located proteIN (EMILIN) gene. J Biol Chem 275, 785–792. 10625608 10.1074/jbc.275.2.785

[34] Leimeister C , Steidl C , Schumacher N , Erhard S & Gessler M (2002) Developmental expression and biochemical characterization of emu family members. Dev Biol 249, 204–218. 12221002 10.1006/dbio.2002.0764

[35] Hao H , Yuan Y , Ito A , Eberand BM , Tjondro H , Cielesh M , Norris N , Moreno CL , Maxwell JWC , Neely GG *et al*. (2025) FUT10 and FUT11 are protein O‐fucosyltransferases that modify protein EMI domains. Nat Chem Biol 21, 598–610. 39775168 10.1038/s41589-024-01815-x PMC11949838

[36] Mongiat M , Mungiguerra G , Bot S , Mucignat MT , Giacomello E , Doliana R & Colombatti A (2000) Self‐assembly and supramolecular organization of EMILIN. J Biol Chem 275, 25471–25480. 10821830 10.1074/jbc.M001426200

[37] Schiavinato A , Becker A‐KA , Zanetti M , Corallo D , Milanetto M , Bizzotto D , Bressan G , Guljelmovic M , Paulsson M , Wagener R *et al*. (2012) EMILIN‐3, peculiar member of elastin microfibril interface‐located protein (EMILIN) family, has distinct expression pattern, forms oligomeric assemblies, and serves as transforming growth factor β (TGF‐β) antagonist. J Biol Chem 287, 11498–11515. 22334695 10.1074/jbc.M111.303578 PMC3322879

[38] Verdone G , Doliana R , Corazza A , Colebrooke SA , Spessotto P , Bot S , Bucciotti F , Capuano A , Silvestri A , Viglino P *et al*. (2008) The solution structure of EMILIN1 globular C1q domain reveals a disordered insertion necessary for interaction with the alpha4beta1 integrin. J Biol Chem 283, 18947–18956. 18463100 10.1074/jbc.M801085200

[39] Braghetta P , Ferrari A , De Gp , Zanetti M , Volpin D , Bonaldo P & Bressan GM (2004) Overlapping, complementary and site‐specific expression pattern of genes of the EMILIN/Multimerin family. Matrix Biol 22, 549–556. 14996434 10.1016/j.matbio.2003.10.005

[40] Bressan GM , ga‐Gordini D , Colombatti A , Castellani I , Marigo V & Volpin D (1993) Emilin, a component of elastic fibers preferentially located at the elastin‐microfibrils interface. J Cell Biol 121, 201–212. 8458869 10.1083/jcb.121.1.201 PMC2119774

[41] Fitoussi R , Beauchef G , Guéré C , André N & Vié K (2019) Localization, fate and interactions of Emilin‐1 in human skin. Int J Cosmet Sci 41, 183–193. 30843221 10.1111/ics.12524

[42] Danussi C , Spessotto P , Petrucco A , Wassermann B , Sabatelli P , Montesi M , Doliana R , Bressan GM & Colombatti A (2008) Emilin1 deficiency causes structural and functional defects of lymphatic vasculature. Mol Cell Biol 28, 4026–4039. 18411305 10.1128/MCB.02062-07 PMC2423131

[43] Danussi C , Petrucco A , Wassermann B , Pivetta E , Modica TM , Del Bel BL , Colombatti A & Spessotto P (2011) EMILIN1‐alpha4/alpha9 integrin interaction inhibits dermal fibroblast and keratinocyte proliferation. J Cell Biol 195, 131–145. 21949412 10.1083/jcb.201008013 PMC3187715

[44] Andreuzzi E , Fejza A , Capuano A , Poletto E , Pivetta E , Doliana R , Pellicani R , Favero A , Maiero S , Fornasarig M *et al*. (2020) Deregulated expression of elastin microfibril Interfacer 2 (EMILIN2) in gastric cancer affects tumor growth and angiogenesis. Matrix Biol Plus 6–7, 100029. 10.1016/j.mbplus.2020.100029 PMC7852313 33543026

[45] Capuano A , Vescovo M , Canesi S , Pivetta E , Doliana R , Nadin MG , Yamamoto M , Tsukamoto T , Nomura S , Pilozzi E *et al*. (2024) The extracellular matrix protein EMILIN‐1 impacts on the microenvironment by hampering gastric cancer development and progression. Gastric Cancer 27, 1016–1030. 38941035 10.1007/s10120-024-01528-z PMC11335817

[46] Schiavinato A , Keene DR , Wohl AP , Corallo D , Colombatti A , Wagener R , Paulsson M , Bonaldo P & Sengle G (2016) Targeting of EMILIN‐1 and EMILIN‐2 to Fibrillin microfibrils facilitates their incorporation into the extracellular matrix. J Invest Dermatol 136, 1150–1160. 26945878 10.1016/j.jid.2016.02.021

[47] Schiavinato A , Marcous F , Zuk AV , Keene DR , Tufa SF , Mosquera LM , Zigrino P , Mauch C , Eckes B , Francois K *et al*. (2024) New insights into the structural role of EMILINs within the human skin microenvironment. Sci Rep 14, 30345. 39639116 10.1038/s41598-024-81509-5 PMC11621341

[48] Paulitti A , Andreuzzi E , Bizzotto D , Pellicani R , Tarticchio G , Marastoni S , Pastrello C , Jurisica I , Ligresti G , Bucciotti F *et al*. (2018) The ablation of the matricellular protein EMILIN2 causes defective vascularization due to impaired EGFR‐dependent IL‐8 production affecting tumor growth. Oncogene 37, 3399–3414. 29483644 10.1038/s41388-017-0107-x

[49] Schiller HB , Fernandez IE , Burgstaller G , Schaab C , Scheltema RA , Schwarzmayr T , Strom TM , Eickelberg O & Mann M (2015) Time‐ and compartment‐resolved proteome profiling of the extracellular niche in lung injury and repair. Mol Syst Biol 11, 819. 26174933 10.15252/msb.20156123 PMC4547847

[50] Van Hoof D , Dormeyer W , Braam SR , Passier R , Monshouwer‐Kloots J , Ward‐van Oostwaard D , Heck AJR , Krijgsveld J & Mummery CL (2010) Identification of cell surface proteins for antibody‐based selection of human embryonic stem cell‐derived cardiomyocytes. J Proteome Res 9, 1610–1618. 20088484 10.1021/pr901138a

[51] Huang M , Sannaningaiah D , Zhao N , Gong Y , Grondolsky J & Hoover‐Plow J (2015) EMILIN2 regulates platelet activation, thrombus formation, and clot retraction. PLoS One 10, e0115284. 25658937 10.1371/journal.pone.0115284 PMC4319747

[52] Sa Q & Hoover‐Plow JL (2011) EMILIN2 (elastin microfibril Interface located protein), potential modifier of thrombosis. Thromb J 9, 9. 21569335 10.1186/1477-9560-9-9 PMC3113922

[53] Letteboer TGW , Benzinou M , Merrick CB , Quigley DA , Zhau K , Kim I‐J , To, MD , Jablons DM , van Amstel JKP , Westermann CJJ *et al*. (2015) Genetic variation in the functional ENG allele inherited from the non‐affected parent associates with presence of pulmonary arteriovenous malformation in hereditary hemorrhagic telangiectasia 1 (HHT1) and may influence expression of PTPN14. Front Genet 6, 67. 25815003 10.3389/fgene.2015.00067 PMC4357294

[54] Amma LL , Goodyear R , Faris JS , Jones I , Ng L , Richardson G & Forrest D (2003) An emilin family extracellular matrix protein identified in the cochlear basilar membrane. Mol Cell Neurosci 23, 460–472. 12837629 10.1016/s1044-7431(03)00075-7

[55] Russell IJ , Lukashkina VA , Levic S , Cho Y‐W , Lukashkin AN , Ng L & Forrest D (2020) Emilin 2 promotes the mechanical gradient of the cochlear basilar membrane and resolution of frequencies in sound. Sci Adv 6, eaba2634. 32577518 10.1126/sciadv.aba2634 PMC7286672

[56] Jean P , Wong Jun Tai F , Singh‐Estivalet A , Lelli A , Scandola C , Megharba S , Schmutz S , Roux S , Mechaussier S , Sudres M *et al*. (2023) Single‐cell transcriptomic profiling of the mouse cochlea: An atlas for targeted therapies. Proc Natl Acad Sci U S A 120, e2221744120. 37339214 10.1073/pnas.2221744120 PMC10293812

[57] Jeimy SB , Krakow EF , Fuller N , Tasneem S & Hayward CPM (2008) An acquired factor V inhibitor associated with defective factor V function, storage and binding to multimerin 1. J Thromb Haemost 6, 395–397. 18047547 10.1111/j.1538-7836.2008.02860.x

[58] Hayward CP , Bainton DF , Smith JW , Horsewood P , Stead RH , Podor TJ , Warkentin TE & Kelton JG (1993) Multimerin is found in the alpha‐granules of resting platelets and is synthesized by a megakaryocytic cell line. J Clin Invest 91, 2630–2639. 8514871 10.1172/JCI116502 PMC443327

[59] Hayward CP , Smith JW , Horsewood P , Warkentin TE & Kelton JG (1991) p‐155, a multimeric platelet protein that is expressed on activated platelets. J Biol Chem 266, 7114–7120. 2016319

[60] Jeimy SB , Woram RA , Fuller N , Quinn‐Allen MA , Nicolaes GAF , Dahlbäck B , Kane WH & Hayward CPM (2004) Identification of the MMRN1 binding region within the C2 domain of human factor V. J Biol Chem 279, 51466–51471. 15452129 10.1074/jbc.M409866200

[61] Hayward CP , Cramer EM , Song Z , Zheng S , Fung R , Massé JM , Stead RH & Podor TJ (1998) Studies of multimerin in human endothelial cells. Blood 91, 1304–1317. 9454761

[62] Sanz‐Moncasi MP , Garin‐Chesa P , Stockert E , Jaffe EA , Old LJ & Rettig WJ (1994) Identification of a high molecular weight endothelial cell surface glycoprotein, endoGlyx‐1, in normal and tumor blood vessels. Lab Invest 71, 366–373. 7933987

[63] Christian S , Ahorn H , Novatchkova M , Garin‐Chesa P , Park JE , Weber G , Eisenhaber F , Rettig WJ & Lenter MC (2001) Molecular cloning and characterization of EndoGlyx‐1, an EMILIN‐like multisubunit glycoprotein of vascular endothelium. J Biol Chem 276, 48588–48595. 11559704 10.1074/jbc.M106152200

[64] Specht CG & Schoepfer R (2004) Deletion of multimerin‐1 in alpha‐synuclein‐deficient mice. Genomics 83, 1176–1178. 15177571 10.1016/j.ygeno.2003.12.014

[65] Pasaje CFA , Bae JS , Park B‐L , Cheong HS , Kim J‐H , Jang A‐S , Uh S‐T , Park C‐S & Shin HD (2012) Possible role of EMID2 on nasal polyps pathogenesis in Korean asthma patients. BMC Med Genet 13, 2. 22217332 10.1186/1471-2350-13-2 PMC3398310

[66] Streuli CH (2009) Integrins and cell‐fate determination. J Cell Sci 122, 171–177. 19118209 10.1242/jcs.018945 PMC2714415

[67] Buraschi S , Neill T & Iozzo RV (2019) Decorin is a devouring proteoglycan: remodeling of intracellular catabolism via autophagy and mitophagy. Matrix Biol 75–76, 260–270. 10.1016/j.matbio.2017.10.005 PMC5938170 29080840

[68] Neill T , Andreuzzi E , Wang Z‐X , Peiper SC , Mongiat M & Iozzo RV (2018) Endorepellin remodels the endothelial transcriptome toward a pro‐autophagic and pro‐mitophagic gene signature. J Biol Chem 293, 12137–12148. 29921586 10.1074/jbc.RA118.002934 PMC6078466

[69] Mongiat M , Pascal G , Poletto E , Williams DM & Iozzo RV (2024) Proteoglycans of basement membranes: crucial controllers of angiogenesis, neurogenesis, and autophagy. Proteoglycan Res 2, e22. 39184370 10.1002/pgr2.22 PMC11340296

[70] Yeger H & Perbal B (2021) The CCN axis in cancer development and progression. J Cell Commun Signal 15, 491–517. 33877533 10.1007/s12079-021-00618-2 PMC8642525

[71] Zacchigna L , Vecchione C , Notte A , Cordenonsi M , Dupont S , Maretto S , Cifelli G , Ferrari A , Maffei A , Fabbro C *et al*. (2006) Emilin1 links TGF‐beta maturation to blood pressure homeostasis. Cell 124, 929–942. 16530041 10.1016/j.cell.2005.12.035

[72] Spessotto P , Cervi M , Mucignat MT , Mungiguerra G , Sartoretto I , Doliana R & Colombatti A (2003) beta 1 integrin‐dependent cell adhesion to EMILIN‐1 is mediated by the gC1q domain. J Biol Chem 278, 6160–6167. 12456677 10.1074/jbc.M208322200

[73] Capuano A , Fogolari F , Bucciotti F , Spessotto P , Nicolosi PA , Mucignat MT , Cervi M , Esposito G , Colombatti A & Doliana R (2018) The α4β1/EMILIN1 interaction discloses a novel and unique integrin‐ligand type of engagement. Matrix Biol 66, 50–66. 29037761 10.1016/j.matbio.2017.10.001

[74] Spessotto P , Bulla R , Danussi C , Radillo O , Cervi M , Monami G , Bossi F , Tedesco F , Doliana R & Colombatti A (2006) EMILIN1 represents a major stromal element determining human trophoblast invasion of the uterine wall. J Cell Sci 119, 4574–4584. 17074837 10.1242/jcs.03232

[75] Danussi C , Del Bel BL , Pivetta E , Modica TM , Muro A , Wassermann B , Doliana R , Sabatelli P , Colombatti A & Spessotto P (2013) EMILIN1/alpha9beta1 integrin interaction is crucial in lymphatic valve formation and maintenance. Mol Cell Biol 33, 4381–4394. 24019067 10.1128/MCB.00872-13 PMC3838180

[76] Danussi C , Petrucco A , Wassermann B , Modica TM , Pivetta E , Del Bel BL , Colombatti A & Spessotto P (2012) An EMILIN1‐negative microenvironment promotes tumor cell proliferation and lymph node invasion. Cancer Prev Res 5, 1131–1143. 10.1158/1940-6207.CAPR-12-0076-T 22827975

[77] Favero A , Segatto I , Capuano A , Mattevi MC , Rampioni Vinciguerra GL , Musco L , D'Andrea S , Dall'Acqua A , Gava C , Perin T *et al*. (2024) Loss of the extracellular matrix glycoprotein EMILIN1 accelerates Δ16HER2‐driven breast cancer initiation in mice. NPJ Breast Cancer 10, 5. 38184660 10.1038/s41523-023-00608-0 PMC10771445

[78] Capuano A , Pivetta E , Sartori G , Bosisio G , Favero A , Cover E , Andreuzzi E , Colombatti A , Cannizzaro R , Scanziani E *et al*. (2019) Abrogation of EMILIN1‐β1 integrin interaction promotes experimental colitis and colon carcinogenesis. Matrix Biol 83, 97–115. 31479698 10.1016/j.matbio.2019.08.006

[79] Modica TME , Maiorani O , Sartori G , Pivetta E , Doliana R , Capuano A , Colombatti A & Spessotto P (2017) The extracellular matrix protein EMILIN1 silences the RAS‐ERK pathway via α4β1 integrin and decreases tumor cell growth. Oncotarget 8, 27034–27046. 28177903 10.18632/oncotarget.15067 PMC5432316

[80] Kamarajan P , Garcia‐Pardo A , D'Silva NJ & Kapila YL (2010) The CS1 segment of fibronectin is involved in human OSCC pathogenesis by mediating OSCC cell spreading, migration, and invasion. BMC Cancer 10, 330. 20579373 10.1186/1471-2407-10-330 PMC3146068

[81] Zucchetto A , Vaisitti T , Benedetti D , Tissino E , Bertagnolo V , Rossi D , Bomben R , Dal Bo M , Del Principe MI , Gorgone A *et al*. (2012) The CD49d/CD29 complex is physically and functionally associated with CD38 in B‐cell chronic lymphocytic leukemia cells. Leukemia 26, 1301–1312. 22289918 10.1038/leu.2011.369

[82] Lund SA , Wilson CL , Raines EW , Tang J , Giachelli CM & Scatena M (2013) Osteopontin mediates macrophage chemotaxis via α4 and α9 integrins and survival via the α4 integrin. J Cell Biochem 114, 1194–1202. 23192608 10.1002/jcb.24462 PMC12462639

[83] Niibori‐Nambu A , Wang CQ , Chin DWL , Chooi JY , Hosoi H , Sonoki T , Tham C‐Y , Nah GSS , Cirovic B , Tan DQ *et al*. (2024) Integrin‐α9 overexpression underlies the niche‐independent maintenance of leukemia stem cells in acute myeloid leukemia. Gene 928, 148761. 39002785 10.1016/j.gene.2024.148761

[84] Tissino E , Pivetta E , Capuano A , Capasso G , Bomben R , Caldana C , Rossi FM , Pozzo F , Benedetti D , Boldorini R *et al*. (2022) Elastin MIcrofibriL INterfacer1 (EMILIN‐1) is an alternative prosurvival VLA‐4 ligand in chronic lymphocytic leukemia. Hematol Oncol 40, 181–190. 34783040 10.1002/hon.2947

[85] Li P‐Y , Lv J , Qi W‐W , Zhao S‐F , Sun L‐B , Liu N , Sheng J & Qiu W‐S (2016) Tspan9 inhibits the proliferation, migration and invasion of human gastric cancer SGC7901 cells via the ERK1/2 pathway. Oncol Rep 36, 448–454. 27177197 10.3892/or.2016.4805

[86] Qi Y , Lv J , Liu S , Sun L , Wang Y , Li H , Qi W & Qiu W (2019) TSPAN9 and EMILIN1 synergistically inhibit the migration and invasion of gastric cancer cells by increasing TSPAN9 expression. BMC Cancer 19, 630. 31242895 10.1186/s12885-019-5810-2 PMC6595627

[87] Sun X , Wang SC , Wei Y , Luo X , Jia Y , Li L , Gopal P , Zhu M , Nassour I , Chuang J‐C *et al*. (2017) Arid1a has context‐dependent oncogenic and tumor suppressor functions in liver cancer. Cancer Cell 32, 574–589. 29136504 10.1016/j.ccell.2017.10.007 PMC5728182

[88] Fan X , Lv C , Xue M , Meng P & Qian X (2024) Fe3O4 nanoparticles containing gambogic acid inhibit metastasis in colorectal cancer via the RORB/EMILIN1 axis. Cell Adh Migr 18, 38–53. 39533963 10.1080/19336918.2024.2427585 PMC11562916

[89] Capuano A , Bucciotti F , Farwell K , Tippin Davis B , Mroske C , Hulick P , Weissman S , Gao Q , Spessotto P , Colombatti A *et al*. (2016) Diagnostic exome sequencing identifies a novel gene, EMILIN1, associated with autosomal‐dominant hereditary connective tissue disease. Hum Mutat 37, 84–97. 26462740 10.1002/humu.22920 PMC4738430

[90] Iacomino M , Doliana R , Marchese M , Capuano A , Striano P , Spessotto P , Bosisio G , Iodice R , Manganelli F , Lanteri P *et al*. (2020) Distal motor neuropathy associated with novel EMILIN1 mutation. Neurobiol Dis 137, 104757. 31978608 10.1016/j.nbd.2020.104757

[91] Maiorani O , Pivetta E , Capuano A , Modica TME , Wassermann B , Bucciotti F , Colombatti A , Doliana R & Spessotto P (2017) Neutrophil elastase cleavage of the gC1q domain impairs the EMILIN1‐α4β1 integrin interaction, cell adhesion and anti‐proliferative activity. Sci Rep 7, 39974. 28074935 10.1038/srep39974 PMC5225433

[92] Pivetta E , Wassermann B , Del Bel Belluz L , Danussi C , Modica TME , Maiorani O , Bosisio G , Boccardo F , Canzonieri V , Colombatti A *et al*. (2016) Local inhibition of elastase reduces EMILIN1 cleavage reactivating lymphatic vessel function in a mouse lymphoedema model. Clin Sci 130, 1221–1236. 10.1042/CS20160064 PMC4888021 26920215

[93] Smith HA & Kang Y (2013) The metastasis‐promoting roles of tumor‐associated immune cells. J Mol Med 91, 411–429. 23515621 10.1007/s00109-013-1021-5 PMC3697909

[94] Dumitru CA , Lang S & Brandau S (2013) Modulation of neutrophil granulocytes in the tumor microenvironment: mechanisms and consequences for tumor progression. Semin Cancer Biol 23, 141–148. 23485549 10.1016/j.semcancer.2013.02.005

[95] Lerman I , Garcia‐Hernandez M d l L , Rangel‐Moreno J , Chiriboga L , Pan C , Nastiuk KL , Krolewski JJ , Sen A & Hammes SR (2017) Infiltrating myeloid cells exert Protumorigenic actions via neutrophil elastase. Mol Cancer Res 15, 1138–1152. 28512253 10.1158/1541-7786.MCR-17-0003 PMC5581693

[96] Papayannopoulos V , Metzler KD , Hakkim A & Zychlinsky A (2010) Neutrophil elastase and myeloperoxidase regulate the formation of neutrophil extracellular traps. J Cell Biol 191, 677–691. 20974816 10.1083/jcb.201006052 PMC3003309

[97] Pivetta E , Danussi C , Wassermann B , Modica TME , Del Bel Belluz L , Canzonieri V , Colombatti A & Spessotto P (2014) Neutrophil elastase‐dependent cleavage compromises the tumor suppressor role of EMILIN1. Matrix Biol 34, 22–32. 24513040 10.1016/j.matbio.2014.01.018

[98] Amor López A , Mazariegos MS , Capuano A , Ximénez‐Embún P , Hergueta‐Redondo M , Recio JÁ , Muñoz E , Al‐Shahrour F , Muñoz J , Megías D *et al*. (2021) Inactivation of EMILIN‐1 by proteolysis and secretion in small extracellular vesicles favors melanoma progression and metastasis. Int J Mol Sci 22, 7406. 34299025 10.3390/ijms22147406 PMC8303474

[99] Deng C , Zhong M‐E , Chen Y , Pan M , Xu L , Xiao Y , Gao Y & Wu B (2023) Proteomic profiling and functional characterization of serum‐derived extracellular vesicles in the mucinous and non‐mucinous colon adenocarcinoma. J Cancer Res Clin Oncol 149, 9285–9300. 37204515 10.1007/s00432-023-04851-7 PMC11797523

[100] Dufresne J , Bowden P , Thavarajah T , Florentinus‐Mefailoski A , Chen ZZ , Tucholska M , Norzin T , Ho MT , Phan M , Mohamed N *et al*. (2019) The plasma peptides of breast versus ovarian cancer. Clin Proteomics 16, 43. 31889940 10.1186/s12014-019-9262-0 PMC6927194

[101] Sun W , Chatterjee B , Wang Y , Stevenson HS , Edelman DC , Meltzer PS & Barr FG (2015) Distinct methylation profiles characterize fusion‐positive and fusion‐negative rhabdomyosarcoma. Mod Pathol 28, 1214–1224. 26226845 10.1038/modpathol.2015.82 PMC6345526

[102] Li J , Xing X , Li D , Zhang B , Mutch DG , Hagemann IS & Wang T (2017) Whole‐genome DNA methylation profiling identifies epigenetic signatures of uterine Carcinosarcoma. Neoplasia 19, 100–111. 28088687 10.1016/j.neo.2016.12.009 PMC5237802

[103] Edlund K , Lindskog C , Saito A , Berglund A , Pontén F , Göransson‐Kultima H , Isaksson A , Jirström K , Planck M , Johansson L *et al*. (2012) CD99 is a novel prognostic stromal marker in non‐small cell lung cancer. Int J Cancer 131, 2264–2273. 22392539 10.1002/ijc.27518

[104] Folgueira MAAK , Carraro DM , Brentani H , Patrão DF d C , Barbosa EM , Netto MM , Caldeira JRF , Katayama MLH , Soares FA , Oliveira CT *et al*. (2005) Gene expression profile associated with response to doxorubicin‐based therapy in breast cancer. Clin Cancer Res 11, 7434–7443. 16243817 10.1158/1078-0432.CCR-04-0548

[105] Rao UNM , Hood BL , Jones‐Laughner JM , Sun M & Conrads TP (2013) Distinct profiles of oxidative stress‐related and matrix proteins in adult bone and soft tissue osteosarcoma and desmoid tumors: a proteomics study. Hum Pathol 44, 725–733. 23063503 10.1016/j.humpath.2012.06.023

[106] Zhang J , Zhang Y , Li Z , Wu H , Xun J & Feng H (2019) Bioinformatics analysis of Ewing's sarcoma: seeking key candidate genes and pathways. Oncol Lett 18, 6008–6016. 31788075 10.3892/ol.2019.10936 PMC6865160

[107] Salani R , Neuberger I , Kurman RJ , Bristow RE , Chang HW , Wang TL & Shih I (2007) Expression of extracellular matrix proteins in ovarian serous tumors. Int J Gynecol Pathol 26, 141–146. 17413980 10.1097/01.pgp.0000229994.02815.f9

[108] Ucaryilmaz Metin C & Ozcan G (2022) Comprehensive bioinformatic analysis reveals a cancer‐associated fibroblast gene signature as a poor prognostic factor and potential therapeutic target in gastric cancer. BMC Cancer 22, 692. 35739492 10.1186/s12885-022-09736-5 PMC9229147

[109] Chen J , Wang X , Hu B , He Y , Qian X & Wang W (2018) Candidate genes in gastric cancer identified by constructing a weighted gene co‐expression network. PeerJ 6, e4692. 29740513 10.7717/peerj.4692 PMC5937478

[110] Formolo CA , Williams R , Gordish‐Dressman H , MacDonald TJ , Lee NH & Hathout Y (2011) Secretome signature of invasive glioblastoma multiforme. J Proteome Res 10, 3149–3159. 21574646 10.1021/pr200210w PMC3136381

[111] Zhao Y , Zhang X , Yao J , Jin Z & Liu C (2020) Expression patterns and the prognostic value of the EMILIN/Multimerin family members in low‐grade glioma. PeerJ 8, e8696. 32175193 10.7717/peerj.8696 PMC7058105

[112] Bunbanjerdsuk S , Vorasan N , Saethang T , Pongrujikorn T , Pangpunyakulchai D , Mongkonsiri N , Arsa L , Thokanit N , Pongsapich W , Anekpuritanang T *et al*. (2019) Oncoproteomic and gene expression analyses identify prognostic biomarkers for second primary malignancy in patients with head and neck squamous cell carcinoma. Mod Pathol 32, 943–956. 30737471 10.1038/s41379-019-0211-2

[113] Reddy RB , Khora SS & Suresh A (2019) Molecular prognosticators in clinically and pathologically distinct cohorts of head and neck squamous cell carcinoma‐a meta‐analysis approach. PLoS One 14, e0218989. 31310629 10.1371/journal.pone.0218989 PMC6634788

[114] Mongiat M , Ligresti G , Marastoni S , Lorenzon E , Doliana R & Colombatti A (2007) Regulation of the extrinsic apoptotic pathway by the extracellular matrix glycoprotein EMILIN2. Mol Cell Biol 27, 7176–7187. 17698584 10.1128/MCB.00696-07 PMC2168889

[115] Marastoni S , Ligresti G , Lorenzon E , Colombatti A & Mongiat M (2008) Extracellular matrix: a matter of life and death. Connect Tissue Res 49, 203–206. 18661343 10.1080/03008200802143190

[116] Mongiat M , Marastoni S , Ligresti G , Lorenzon E , Schiappacassi M , Perris R , Frustaci S & Colombatti A (2010) The extracellular matrix glycoprotein elastin microfibril interface located protein 2: a dual role in the tumor microenvironment. Neoplasia 12, 294–304. 20360940 10.1593/neo.91930 PMC2847737

[117] Marastoni S , Andreuzzi E , Paulitti A , Colladel R , Pellicani R , Todaro F , Schiavinato A , Bonaldo P , Colombatti A & Mongiat M (2014) EMILIN2 down‐modulates the Wnt signalling pathway and suppresses breast cancer cell growth and migration. J Pathol 232, 391–404. 24374807 10.1002/path.4316

[118] Broniarczyk J , Trejo‐Cerro O , Massimi P , Kavčič N , Myers MP & Banks L (2024) HPV‐18 E6 enhances the interaction between EMILIN2 and SNX27 to promote WNT signaling. J Virol 98, e0073524. 38874360 10.1128/jvi.00735-24 PMC11265340

[119] Wisztorski M , Aboulouard S , Roussel L , Duhamel M , Saudemont P , Cardon T , Narducci F , Robin Y‐M , Lemaire A‐S , Bertin D *et al*. (2023) Fallopian tube lesions as potential precursors of early ovarian cancer: a comprehensive proteomic analysis. Cell Death Dis 14, 644. 37775701 10.1038/s41419-023-06165-5 PMC10541450

[120] Tang X & Li F (2022) Decreased EMILIN2 correlates to metabolism phenotype and poor prognosis of ovarian cancer. J Biochem 172, 89–97. 35588228 10.1093/jb/mvac046

[121] Tao R , Li J , Xin J , Wu J , Guo J , Zhang L , Jiang L , Zhang W , Yang Z & Li L (2011) Methylation profile of single hepatocytes derived from hepatitis B virus‐related hepatocellular carcinoma. PLoS One 6, e19862. 21625442 10.1371/journal.pone.0019862 PMC3100314

[122] Hill VK , Hesson LB , Dansranjavin T , Dallol A , Bieche I , Vacher S , Tommasi S , Dobbins T , Gentle D , Euhus D *et al*. (2010) Identification of 5 novel genes methylated in breast and other epithelial cancers. Mol Cancer 9, 51. 20205715 10.1186/1476-4598-9-51 PMC2841122

[123] Tessema M , Yingling CM , Liu Y , Tellez CS , Van NL , Baylin SS & Belinsky SA (2014) Genome‐wide unmasking of epigenetically silenced genes in lung adenocarcinoma from smokers and never smokers. Carcinogenesis 35, 1248–1257. 24398667 10.1093/carcin/bgt494 PMC4110480

[124] Andreuzzi E , Fejza A , Polano M , Poletto E , Camicia L , Carobolante G , Tarticchio G , Todaro F , Di Carlo E , Scarpa M *et al*. (2022) Colorectal cancer development is affected by the ECM molecule EMILIN‐2 hinging on macrophage polarization via the TLR‐4/MyD88 pathway. J Exp Clin Cancer Res 41, 60. 35148799 10.1186/s13046-022-02271-y PMC8840294

[125] Scholler N , Gross JA , Garvik B , Wells L , Liu Y , Loch CM , Ramirez AB , McIntosh MW , Lampe PD & Urban N (2008) Use of cancer‐specific yeast‐secreted in vivo biotinylated recombinant antibodies for serum biomarker discovery. J Transl Med 6, 41. 18652693 10.1186/1479-5876-6-41 PMC2503970

[126] Fejza A , Polano M , Camicia L , Poletto E , Carobolante G , Toffoli G , Mongiat M & Andreuzzi E (2021) The efficacy of anti‐PD‐L1 treatment in melanoma is associated with the expression of the ECM molecule EMILIN2. Int J Mol Sci 22, 7511. 34299131 10.3390/ijms22147511 PMC8306837

[127] Mann B , Madera M , Klouckova I , Mechref Y , Dobrolecki LE , Hickey RJ , Hammoud ZT & Novotny MV (2010) A quantitative investigation of fucosylated serum glycoproteins with application to esophageal adenocarcinoma. Electrophoresis 31, 1833–1841. 20446296 10.1002/elps.201000046 PMC3078823

[128] Dadone‐Montaudié B , Alberti L , Duc A , Delespaul L , Lesluyes T , Pérot G , Lançon A , Paindavoine S , Di Mauro I , Blay J‐Y *et al*. (2018) Alternative PDGFD rearrangements in dermatofibrosarcomas protuberans without PDGFB fusions. Mod Pathol 31, 1683–1693. 29955147 10.1038/s41379-018-0089-4

[129] Lee P‐H , Huang S‐C , Wu P‐S , Tai H‐C , Lee C‐H , Lee J‐C , Kao Y‐C , Tsai J‐W , Hsieh T‐H , Li C‐F *et al*. (2022) Molecular characterization of dermatofibrosarcoma protuberans: the Clinicopathologic significance of uncommon fusion gene rearrangements and their diagnostic importance in the exclusively subcutaneous and circumscribed lesions. Am J Surg Pathol 46, 942–955. 35034038 10.1097/PAS.0000000000001866

[130] Chandler B , Jing F , David MP & Nazarullah A (2023) Platelet‐derived growth factor‐D fusion‐positive dermatofibrosarcoma protuberans: case report of an atypical breast mass and literature review. Int J Surg Pathol 31, 1610–1617. 37016743 10.1177/10668969231160261

[131] Zhao G , Zheng J , Tang K & Chen Q (2022) EMILIN2 is associated with prognosis and immunotherapy in clear cell renal cell carcinoma. Front Genet 13, 1058207. 36544490 10.3389/fgene.2022.1058207 PMC9760906

[132] Warnecke‐Eberz U , Metzger R , Holscher AH , Drebber U & Bollschweiler E (2016) Diagnostic marker signature for esophageal cancer from transcriptome analysis. Tumour Biol 37, 6349–6358. 26631031 10.1007/s13277-015-4400-4

[133] Wang L‐C , Cui W‐Y , Zhang Z , Tan Z‐L , Lv Q‐L , Chen S‐H & Shen X‐L (2021) Expression, methylation and prognostic feature of EMILIN2 in low‐grade‐glioma. Brain Res Bull 175, 26–36. 34280481 10.1016/j.brainresbull.2021.07.013

[134] Wang LA , Zheng Z , Zheng J , Zhang G & Wang Z (2024) The potential significance of the EMILIN3 gene in augmenting the aggressiveness of low‐grade gliomas is noteworthy. Cancer Manag Res 16, 711–730. 38952353 10.2147/CMAR.S463694 PMC11215280

[135] Zeng W‐J , Yang Y‐L , Liu Z‐Z , Wen Z‐P , Chen Y‐H , Hu X‐L , Cheng Q , Xiao J , Zhao J & Chen X‐P (2018) Integrative analysis of DNA methylation and gene expression identify a three‐gene signature for predicting prognosis in lower‐grade gliomas. Cell Physiol Biochem 47, 428–439. 29794476 10.1159/000489954

[136] Liu H‐Q , Li W‐X , An Y‐W , Wu T , Jiang G‐Y , Dong Y , Chen W‐X , Wang J‐C , Wang C & Song S (2022) Integrated analysis of the genomic and transcriptional profile of gliomas with isocitrate dehydrogenase‐1 and tumor protein 53 mutations. Int J Immunopathol Pharmacol 36, 3946320221139262. 36377597 10.1177/03946320221139262 PMC9669701

[137] Ali Hassan NZ , Mokhtar NM , Kok Sin T , Mohamed Rose I , Sagap I , Harun R & Jamal R (2014) Integrated analysis of copy number variation and genome‐wide expression profiling in colorectal cancer tissues. PLoS One 9, e92553. 24694993 10.1371/journal.pone.0092553 PMC3973632

[138] Michl M , Taverna F , Woischke C , Li P , Klauschen F , Kirchner T , Heinemann V , von Bergwelt‐Baildon M , Stahler A , Herold TM *et al*. (2024) Identification of a gene expression signature associated with brain metastasis in colorectal cancer. Clin Transl Oncol 26, 1886–1895. 38558282 10.1007/s12094-024-03408-5 PMC11249597

[139] Tomasi F , Pozzi M & Lauria M (2024) Investigating the mechanisms underlying resistance to chemotherapy and to CRISPR‐Cas9 in cancer cell lines. Sci Rep 14, 5402. 38443409 10.1038/s41598-024-55138-x PMC10915165

[140] Leatherdale A , Parker D , Tasneem S , Wang Y , Bihan D , Bonna A , Hamaia SW , Gross PL , Ni H , Doble BW *et al*. (2021) Multimerin 1 supports platelet function in vivo and binds to specific GPAGPOGPX motifs in fibrillar collagens that enhance platelet adhesion. J Thromb Haemost 19, 547–561. 33179420 10.1111/jth.15171 PMC7898486

[141] Li S , Lu Z , Wu S , Chu T , Li B , Qi F , Zhao Y & Nie G (2024) The dynamic role of platelets in cancer progression and their therapeutic implications. Nat Rev Cancer 24, 72–87. 38040850 10.1038/s41568-023-00639-6

[142] Engel J (1989) EGF‐like domains in extracellular matrix proteins: localized signals for growth and differentiation? FEBS Lett 251, 1–7. 2666164 10.1016/0014-5793(89)81417-6

[143] Tombling BJ , Wang CK & Craik DJ (2020) EGF‐like and other disulfide‐rich microdomains as therapeutic scaffolds. Angew Chem Int Ed Engl 59, 11218–11232. 31867866 10.1002/anie.201913809

[144] Posner MG (2022) Multimerin‐1 and cancer: a review. Biosci Rep 42, BSR20211248. 35132992 10.1042/BSR20211248 PMC8881648

[145] Martinez I , Wang J , Hobson KF , Ferris RL & Khan SA (2007) Identification of differentially expressed genes in HPV‐positive and HPV‐negative oropharyngeal squamous cell carcinomas. Eur J Cancer 43, 415–432. 17079134 10.1016/j.ejca.2006.09.001 PMC1847595

[146] Tsai T‐H , Song E , Zhu R , Di Poto C , Wang M , Luo Y , Varghese RS , Tadesse MG , Ziada DH , Desai CS *et al*. (2015) LC‐MS/MS‐based serum proteomics for identification of candidate biomarkers for hepatocellular carcinoma. Proteomics 15, 2369–2381. 25778709 10.1002/pmic.201400364 PMC4490019

[147] Laszlo GS , Alonzo TA , Gudgeon CJ , Harrington KH , Gerbing RB , Wang Y‐C , Ries RE , Raimondi SC , Hirsch BA , Gamis AS *et al*. (2015) Multimerin‐1 (MMRN1) as novel adverse marker in pediatric acute myeloid leukemia: a report from the Children's oncology group. Clin Cancer Res 21, 3187–3195. 25825478 10.1158/1078-0432.CCR-14-2684 PMC4506237

[148] Pan S , Chen R , Crispin DA , May D , Stevens T , McIntosh MW , Bronner MP , Ziogas A , Anton‐Culver H & Brentnall TA (2011) Protein alterations associated with pancreatic cancer and chronic pancreatitis found in human plasma using global quantitative proteomics profiling. J Proteome Res 10, 2359–2376. 21443201 10.1021/pr101148r PMC3090497

[149] Chokchaichamnankit D , Watcharatanyatip K , Subhasitanont P , Weeraphan C , Keeratichamroen S , Sritana N , Kantathavorn N , Diskul‐Na‐Ayudthaya P , Saharat K , Chantaraamporn J *et al*. (2019) Urinary biomarkers for the diagnosis of cervical cancer by quantitative label‐free mass spectrometry analysis. Oncol Lett 17, 5453–5468. 31186765 10.3892/ol.2019.10227 PMC6507435

[150] Saini A , Kumar V , Tomar AK , Sharma A & Yadav S (2023) Multimerin 1 aids in the progression of ovarian cancer possibly via modulation of DNA damage response and repair pathways. Mol Cell Biochem 478, 2395–2403. 36723821 10.1007/s11010-023-04668-5

[151] Liu M , Hu P , Tang B , Yang Q , Xiang R , Liu Y , Li J , Wu B , Wu H , Tian B *et al*. (2024) Endoplasmic reticulum stress‐MMRN1 positive feedback contributes to cisplatin resistance in small cell lung cancer. J Thorac Dis 16, 8363–8378. 39831245 10.21037/jtd-24-1477 PMC11740035

[152] Saini A , Chandra KB , Kumar V , Mathur SR , Sharma JB , Kumar S & Yadav S (2020) Analysis of Multimerin 1 (MMRN1) expression in ovarian cancer. Mol Biol Rep 47, 9459–9468. 33263168 10.1007/s11033-020-06027-9

[153] Tajmul M , Parween F , Singh L , Mathur SR , Sharma JB , Kumar S , Sharma DN & Yadav S (2018) Identification and validation of salivary proteomic signatures for non‐invasive detection of ovarian cancer. Int J Biol Macromol 108, 503–514. 29222021 10.1016/j.ijbiomac.2017.12.014

[154] Zhang H‐T , Tian E‐B , Chen Y‐L , Deng H‐T & Wang Q‐T (2015) Proteomic analysis for finding serum pathogenic factors and potential biomarkers in multiple myeloma. Chin Med J 128, 1108–1113. 25881608 10.4103/0366-6999.155112 PMC4832954

[155] Yin X , Subramanian S , Hwang S‐J , O'Donnell CJ , Fox CS , Courchesne P , Muntendam P , Gordon N , Adourian A , Juhasz P *et al*. (2014) Protein biomarkers of new‐onset cardiovascular disease: prospective study from the systems approach to biomarker research in cardiovascular disease initiative. Arterioscler Thromb Vasc Biol 34, 939–945. 24526693 10.1161/ATVBAHA.113.302918 PMC4061732

[156] Saygin D , Tabib T , Bittar HET , Valenzi E , Sembrat J , Chan SY , Rojas M & Lafyatis R (2020) Transcriptional profiling of lung cell populations in idiopathic pulmonary arterial hypertension. Pulm Circ 10, 1–15. 10.1177/2045894020908782 PMC7052475 32166015

[157] Baquet‐Walscheid K , Wildschütz L , Kasper M , Busch M , Thanos S , Bauer D , Stoll M , König S & Heiligenhaus A (2022) Assessment of angiogenesis‐related parameters in juvenile idiopathic arthritis‐associated uveitis. Mol Biol Rep 49, 6093–6102. 35359237 10.1007/s11033-022-07398-x

[158] Amati F , Vancheri C , Latini A , Colona VL , Grelli S , D'Apice MR , Balestrieri E , Passarelli C , Minutolo A , Loddo S *et al*. (2020) Expression profiles of the SARS‐CoV‐2 host invasion genes in nasopharyngeal and oropharyngeal swabs of COVID‐19 patients. Heliyon 6, e05143. 33024851 10.1016/j.heliyon.2020.e05143 PMC7528978

[159] Dulski J , Uitti RJ , Beasley A , Hernandez D , Ramanan VK , Cahn EJ , Ren Y , Johnson PW , Quicksall ZS , Wszolek ZK *et al*. (2024) Genetics of Parkinson's disease heterogeneity: a genome‐wide association study of clinical subtypes. Parkinsonism Relat Disord 119, 105935. 38072719 10.1016/j.parkreldis.2023.105935 PMC10872335

[160] Bora A , Ubaida Mohien C , Chaerkady R , Chang L , Moxley R , Sacktor N , Haughey N , McArthur JC , Cotter R , Nath A *et al*. (2014) Identification of putative biomarkers for HIV‐associated neurocognitive impairment in the CSF of HIV‐infected patients under cART therapy determined by mass spectrometry. J Neurovirol 20, 457–465. 25056907 10.1007/s13365-014-0263-5 PMC4493746

[161] Zheng W , Zhang S , Guo H , Chen X , Huang Z , Jiang S & Li M (2021) Multi‐omics analysis of tumor angiogenesis characteristics and potential epigenetic regulation mechanisms in renal clear cell carcinoma. Cell Commun Signal 19, 39. 33761933 10.1186/s12964-021-00728-9 PMC7992844

[162] Cho H‐J , Lee S , Ji YG & Lee DH (2018) Association of specific gene mutations derived from machine learning with survival in lung adenocarcinoma. PLoS One 13, e0207204. 30419062 10.1371/journal.pone.0207204 PMC6231670

[163] Andreuzzi E , Colladel R , Pellicani R , Tarticchio G , Cannizzaro R , Spessotto P , Bussolati B , Brossa A , De PP , Canzonieri V *et al*. (2017) The angiostatic molecule Multimerin 2 is processed by MMP‐9 to allow sprouting angiogenesis. Matrix Biol 64, 40–53. 28435016 10.1016/j.matbio.2017.04.002

[164] Andreuzzi E , Capuano A , Pellicani R , Poletto E , Doliana R , Maiero S , Fornasarig M , Magris R , Colombatti A , Cannizzaro R *et al*. (2018) Loss of Multimerin‐2 and EMILIN‐2 expression in gastric cancer associate with altered angiogenesis. Int J Mol Sci 19, 3983. 30544909 10.3390/ijms19123983 PMC6321373

[165] Capuano A , Andreuzzi E , Pivetta E , Doliana R , Favero A , Canzonieri V , Maiero S , Fornasarig M , Magris R , Cannizzaro R *et al*. (2019) The probe based confocal laser Endomicroscopy (pCLE) in locally advanced gastric cancer: a powerful technique for real‐time analysis of vasculature. Front Oncol 9, 513. 31263680 10.3389/fonc.2019.00513 PMC6584847

[166] Shield‐Artin KL , Bailey MJ , Oliva K , Liovic AK , Barker G , Dellios NL , Reisman S , Ayhan M & Rice GE (2012) Identification of ovarian cancer‐associated proteins in symptomatic women: a novel method for semi‐quantitative plasma proteomics. Proteomics Clin Appl 6, 170–181. 22532453 10.1002/prca.201100008

[167] Soltermann A , Ossola R , Kilgus‐Hawelski S , von EA , Suter T , Aebersold R & Moch H (2008) N‐glycoprotein profiling of lung adenocarcinoma pleural effusions by shotgun proteomics. Cancer 114, 124–133. 18327805 10.1002/cncr.23349

[168] Beretov J , Wasinger VC , Millar EKA , Schwartz P , Graham PH & Li Y (2015) Proteomic analysis of urine to identify breast cancer biomarker candidates using a label‐free LC‐MS/MS approach. PLoS One 10, e0141876. 26544852 10.1371/journal.pone.0141876 PMC4636393

[169] Cao Z , Zhang Z , Tang X , Liu R , Wu M , Wu J & Liu Z (2022) Comprehensive analysis of tissue proteomics in patients with papillary thyroid microcarcinoma uncovers the underlying mechanism of lymph node metastasis and its significant sex disparities. Front Oncol 12, 887977. 36106120 10.3389/fonc.2022.887977 PMC9465038

[170] Liu H , Zhang J , Zhao Y , Fan Z , Yang Y , Mao Y , Yang J & Ma S (2024) CD93 regulates breast cancer growth and vasculogenic mimicry through the PI3K/AKT/SP2 signaling pathway activated by integrin β1. J Biochem Mol Toxicol 38, e23688. 38511888 10.1002/jbt.23688

[171] Kawata T , Muramatsu K , Shishito N , Ichikawa‐Tomikawa N , Oishi T , Kakuda Y , Akiyama Y , Yamaguchi K , Sakamoto M & Sugino T (2021) EMID1, a multifunctional molecule identified in a murine model for the invasion independent metastasis pathway. Sci Rep 11, 16372. 34385585 10.1038/s41598-021-96006-2 PMC8361151

[172] Qin S , Wei T , Mo J , Lu L , Chai X , Huang Q , Qi S & Tan G (2024) Research on the shared function of central neurons and breast cancer based on gene expression profile data mining: the role of EMID1 protein antibody expression. Int J Biol Macromol 277, 134393. 39094856 10.1016/j.ijbiomac.2024.134393

[173] Shao Y , Zheng Z , Li S , Yang G , Qi F & Fei F (2022) Upregulation of EMID1 accelerates to a favorable prognosis and immune infiltration in lung adenocarcinoma. J Oncol 2022, 5185202. 36245990 10.1155/2022/5185202 PMC9553514

[174] Larson SR , Zhang X , Dumpit R , Coleman I , Lakely B , Roudier M , Higano CS , True LD , Lange PH , Montgomery B *et al*. (2013) Characterization of osteoblastic and osteolytic proteins in prostate cancer bone metastases. Prostate 73, 932–940. 23334979 10.1002/pros.22639 PMC4214278

[175] Cappelletto A , Alfì E , Volf N , Vu TVA , Bortolotti F , Ciucci G , Vodret S , Fantuz M , Perin M , Colliva A *et al*. (2024) EMID2 is a novel biotherapeutic for aggressive cancers identified by in vivo screening. J Exp Clin Cancer Res 43, 15. 38195652 10.1186/s13046-023-02942-4 PMC10777502

[176] Capuano A , Pivetta E , Baldissera F , Bosisio G , Wassermann B , Bucciotti F , Colombatti A , Sabatelli P , Doliana R & Spessotto P (2019) Integrin binding site within the gC1q domain orchestrates EMILIN‐1‐induced lymphangiogenesis. Matrix Biol 81, 34–49. 30408617 10.1016/j.matbio.2018.10.006

[177] Petrova TV & Koh GY (2020) Biological functions of lymphatic vessels. Science 369, eaax4063. 32646971 10.1126/science.aax4063

[178] Pivetta E , Capuano A , Vescovo M , Scanziani E , Cappelleri A , Rampioni Vinciguerra GL , Vecchione A , Doliana R , Mongiat M & Spessotto P (2022) EMILIN‐1 deficiency promotes chronic inflammatory disease through TGFβ signaling alteration and impairment of the gC1q/α4β1 integrin interaction. Matrix Biol 111, 133–152. 35764213 10.1016/j.matbio.2022.06.005

[179] Honda CK , Kurozumi S , Fujii T , Pourquier D , Khellaf L , Boissiere F , Horiguchi J , Oyama T , Shirabe K , Colinge J *et al*. (2024) Cancer‐associated fibroblast spatial heterogeneity and EMILIN1 expression in the tumor microenvironment modulate TGF‐β activity and CD8+ T‐cell infiltration in breast cancer. Theranostics 14, 1873–1885. 38505604 10.7150/thno.90627 PMC10945331

[180] Lai J , Huang R & Huang J (2025) Stemness‐related gene signatures as a predictive tool for breast cancer radiosensitivity. Front Immunol 16, 1536284. 39958347 10.3389/fimmu.2025.1536284 PMC11825753

[181] Fejza A , Camicia L , Carobolante G , Poletto E , Paulitti A , Schinello G , Di Siena E , Cannizzaro R , Iozzo RV , Baldassarre G *et al*. (2023) Emilin2 fosters vascular stability by promoting pericyte recruitment. Matrix Biol 122, 18–32. 37579864 10.1016/j.matbio.2023.08.002

[182] Da Ros F , Persano L , Bizzotto D , Michieli M , Braghetta P , Mazzucato M & Bonaldo P (2022) Emilin‐2 is a component of bone marrow extracellular matrix regulating mesenchymal stem cell differentiation and hematopoietic progenitors. Stem Cell Res Ther 13, 2. 35012633 10.1186/s13287-021-02674-2 PMC8744352

[183] Geng Z , Liu J , Hu J , Wang Y , Tao Y , Zheng F , Wang Y , Fu S , Wang W , Xie C *et al*. (2020) Crucial transcripts predict response to initial immunoglobulin treatment in acute Kawasaki disease. Sci Rep 10, 17860. 33082496 10.1038/s41598-020-75039-z PMC7575539

[184] Plubell DL , Wilmarth PA , Zhao Y , Fenton AM , Minnier J , Reddy AP , Klimek J , Yang X , David LL & Pamir N (2017) Extended multiplexing of tandem mass tags (TMT) labeling reveals age and high fat diet specific proteome changes in mouse Epididymal adipose tissue. Mol Cell Proteomics 16, 873–890. 28325852 10.1074/mcp.M116.065524 PMC5417827

[185] Haage V , Semtner M , Vidal RO , Hernandez DP , Pong WW , Chen Z , Hambardzumyan D , Magrini V , Ly A , Walker J *et al*. (2019) Comprehensive gene expression meta‐analysis identifies signature genes that distinguish microglia from peripheral monocytes/macrophages in health and glioma. Acta Neuropathol Commun 7, 20. 30764877 10.1186/s40478-019-0665-y PMC6376799

[186] Liu G , Wang L , Ji L , He D , Zeng L , Zhuo G , Zhang Q , Wang D & Pan Y (2023) Identifying prognostic markers in spatially heterogeneous breast cancer microenvironment. J Transl Med 21, 580. 37644433 10.1186/s12967-023-04395-x PMC10463390

[187] Huijbers EJM , van Beijnum JR , Loon K , Griffioen CJ , Volckmann R , Bassez A , Lambrechts D , Nunes Monteiro M , Jimenez CR , Hogendoorn PCW *et al*. (2025) Embryonic reprogramming of the tumor vasculature reveals targets for cancer therapy. Proc Natl Acad Sci USA 122, e2424730122. 40096611 10.1073/pnas.2424730122 PMC11962416

[188] Derynck R , Akhurst RJ & Balmain A (2001) TGF‐beta signaling in tumor suppression and cancer progression. Nat Genet 29, 117–129. 11586292 10.1038/ng1001-117

[189] Corallo D , Schiavinato A , Trapani V , Moro E , Argenton F & Bonaldo P (2013) Emilin3 is required for notochord sheath integrity and interacts with Scube2 to regulate notochord‐derived hedgehog signals. Development 140, 4594–4601. 24131633 10.1242/dev.094078

[190] Corallo D , Schiavinato A , Bizzotto D , Milanetto M , Guljelmovic M , Keene DR , Sengle G , Braghetta P & Bonaldo P (2017) EMILIN3, an extracellular matrix molecule with restricted distribution in skin. Exp Dermatol 26, 435–438. 27892605 10.1111/exd.13254

[191] Sheets AR , Demidova‐Rice TN , Shi L , Ronfard V , Grover KV & Herman IM (2016) Identification and characterization of novel matrix‐derived bioactive peptides: a role for collagenase from Santyl® ointment in post‐debridement wound healing? PLoS One 11, e0159598. 27459729 10.1371/journal.pone.0159598 PMC4961374

[192] Brophy TM , Coller BS & Ahamed J (2013) Identification of the thiol isomerase‐binding peptide, mastoparan, as a novel inhibitor of shear‐induced transforming growth factor β1 (TGF‐β1) activation. J Biol Chem 288, 10628–10639. 23463512 10.1074/jbc.M112.439034 PMC3624443

[193] Adam F , Zheng S , Joshi N , Kelton DS , Sandhu A , Suehiro Y , Jeimy SB , Santos AV , Massé J‐M , Kelton JG *et al*. (2005) Analyses of cellular multimerin 1 receptors: in vitro evidence of binding mediated by alphaIIbbeta3 and alphavbeta3. Thromb Haemost 94, 1004–1011. 16363244 10.1160/TH05-02-0140

[194] Tasneem S , Adam F , Minullina I , Pawlikowska M , Hui SK , Zheng S , Miller JL & Hayward CPM (2009) Platelet adhesion to multimerin 1 in vitro: influences of platelet membrane receptors, von Willebrand factor and shear. J Thromb Haemost 7, 685–692. 19175495 10.1111/j.1538-7836.2009.03284.x

[195] Lorenzon E , Colladel R , Andreuzzi E , Marastoni S , Todaro F , Schiappacassi M , Ligresti G , Colombatti A & Mongiat M (2012) MULTIMERIN2 impairs tumor angiogenesis and growth by interfering with VEGF‐A/VEGFR2 pathway. Oncogene 31, 3136–3147. 22020326 10.1038/onc.2011.487

[196] Colladel R , Pellicani R , Andreuzzi E , Paulitti A , Tarticchio G , Todaro F , Colombatti A & Mongiat M (2016) MULTIMERIN2 binds VEGF‐A primarily via the carbohydrate chains exerting an angiostatic function and impairing tumor growth. Oncotarget 7, 2022–2037. 26655500 10.18632/oncotarget.6515 PMC4811514

[197] Pellicani R , Poletto E , Andreuzzi E , Paulitti A , Doliana R , Bizzotto D , Braghetta P , Colladel R , Tarticchio G , Sabatelli P *et al*. (2020) Multimerin‐2 maintains vascular stability and permeability. Matrix Biol 87, 11–25. 31422156 10.1016/j.matbio.2019.08.002

[198] Khan KA , McMurray JL , Mohammed F & Bicknell R (2019) C‐type lectin domain group 14 proteins in vascular biology, cancer and inflammation. FEBS J 286, 3299–3332. 31287944 10.1111/febs.14985 PMC6852297

[199] Neag G , Lewis J , Turner JD , Manning JE , Dean I , Finlay M , Poologasundarampillai G , Woods J , Sahu MA , Khan KA *et al*. (2024) Type‐H endothelial cell protein Clec14a orchestrates osteoblast activity during trabecular bone formation and patterning. Commun Biol 7, 1296. 39394430 10.1038/s42003-024-06971-3 PMC11470016

[200] Cipriani P , Ruscitti P , Di Cola I , Vomero M , Abbruzzese F , Di Nino E , Ross R , Del Galdo F & Giacomelli R (2023) Fibroblast expression of CD248 may contribute to exacerbation of microvascular damage during systemic sclerosis. Rheumatology 62, 1317–1325. 35916713 10.1093/rheumatology/keac377

[201] Liang Q , Su L , Zhang D & Jiao J (2020) CD93 negatively regulates astrogenesis in response to MMRN2 through the transcriptional repressor ZFP503 in the developing brain. Proc Natl Acad Sci USA 117, 9413–9422. 32291340 10.1073/pnas.1922713117 PMC7196765

[202] Zanivan S , Maione F , Hein MY , Hernandez‐Fernaud JR , Ostasiewicz P , Giraudo E & Mann M (2013) SILAC‐based proteomics of human primary endothelial cell morphogenesis unveils tumor Angiogenic markers. Mol Cell Proteomics 12, 3599–3611. 23979707 10.1074/mcp.M113.031344 PMC3861710

[203] Galvagni F , Nardi F , Spiga O , Trezza A , Tarticchio G , Pellicani R , Andreuzzi E , Caldi E , Toti P , Tosi GM *et al*. (2017) Dissecting the CD93‐Multimerin 2 interaction involved in cell adhesion and migration of the activated endothelium. Matrix Biol 64, 112–127. 28912033 10.1016/j.matbio.2017.08.003

[204] Khan KA , Naylor AJ , Khan A , Noy PJ , Mambretti M , Lodhia P , Athwal J , Korzystka A , Buckley CD , Willcox BE *et al*. (2017) Multimerin‐2 is a ligand for group 14 family C‐type lectins CLEC14A, CD93 and CD248 spanning the endothelial pericyte interface. Oncogene 36, 6097–6108. 28671670 10.1038/onc.2017.214 PMC5671938

[205] Barbera S , Raucci L , Tassone G , Tinti L , Prischi F , Santucci A , Mongiat M , Tosi GM , Galvagni F , Dimberg A *et al*. (2023) Dimerization of the C‐type lectin‐like receptor CD93 promotes its binding to Multimerin‐2 in endothelial cells. Int J Biol Macromol 224, 453–464. 36265539 10.1016/j.ijbiomac.2022.10.136

[206] Noy PJ , Lodhia P , Khan K , Zhuang X , Ward DG , Verissimo AR , Bacon A & Bicknell R (2015) Blocking CLEC14A‐MMRN2 binding inhibits sprouting angiogenesis and tumour growth. Oncogene 34, 5821–5831. 25745997 10.1038/onc.2015.34 PMC4724939

[207] Cicaloni V , Karmakar M , Frusciante L , Pettini F , Visibelli A , Orlandini M , Galvagni F , Mongiat M , Silk M , Nardi F *et al*. (2022) Bioinformatics approaches to predict mutation effects in the binding site of the proangiogenic molecule CD93. Front Bioinform 2, 891553. 36353214 10.3389/fbinf.2022.891553 PMC9638713

[208] Lugano R , Vemuri K , Yu D , Bergqvist M , Smits A & Essand M (2018) CD93 promotes β1 integrin activation and fibronectin fibrillogenesis during tumor angiogenesis. J Clin Invest 128, 3280–3297. 29763414 10.1172/JCI97459 PMC6063507

[209] Barbera S , Nardi F , Elia I , Realini G , Lugano R , Santucci A , Tosi GM , Dimberg A , Galvagni F & Orlandini M (2019) The small GTPase Rab5c is a key regulator of trafficking of the CD93/Multimerin‐2/β1 integrin complex in endothelial cell adhesion and migration. Cell Commun Signal 17, 55. 31138217 10.1186/s12964-019-0375-x PMC6537425

[210] Vemuri K , de Alves Pereira B , Fuenzalida P , Subashi Y , Barbera S , van Hooren L , Hedlund M , Pontén F , Lindskog C , Olsson A‐K *et al*. (2024) CD93 maintains endothelial barrier function and limits metastatic dissemination. JCI Insight 9, e169830. 38441970 10.1172/jci.insight.169830 PMC11128212

[211] Raucci L , Perrone CD , Barbera S , de Boer LJ , Tosi GM , Brunetti J , Bracci L , Pozzi C , Galvagni F & Orlandini M (2025) Structural and antigen‐binding surface definition of an anti‐CD93 monoclonal antibody for the treatment of degenerative vascular eye diseases. Int J Biol Macromol 309, 143118. 40228767 10.1016/j.ijbiomac.2025.143118

[212] Liang Y , Zhang S , Wang D , Ji P , Zhang B , Wu P , Wang L , Liu Z , Wang J , Duan Y *et al*. (2024) Dual‐functional Nanodroplet for tumor vasculature ultrasound imaging and tumor immunosuppressive microenvironment remodeling. Adv Healthc Mater 13, e2401274. 39031111 10.1002/adhm.202401274

[213] Valdez Y , Maia M & Conway EM (2012) CD248: reviewing its role in health and disease. Curr Drug Targets 13, 432–439. 22206249 10.2174/138945012799424615

[214] Fejza A , Poletto E , Carobolante G , Camicia L , Andreuzzi E , Capuano A , Pivetta E , Pellicani R , Colladel R , Marastoni S *et al*. (2021) Multimerin‐2 orchestrates the cross‐talk between endothelial cells and pericytes: a mechanism to maintain vascular stability. Matrix Biol Plus 11, 100068. 34435184 10.1016/j.mbplus.2021.100068 PMC8377000

[215] Wang X , Cui Z , Chen X , Luo Q , Jiang Z , Liu X , Huang Y , Jiang J , Chen S , Qiu J *et al*. (2023) The CXCR4/miR‐1910‐5p/MMRN2 Axis is involved in corneal neovascularization by affecting vascular permeability. Invest Ophthalmol Vis Sci 64, 10. 10.1167/iovs.64.4.10 PMC10108737 37040097

[216] Rueda A , Serna N , Mangues R , Villaverde A & Unzueta U (2025) Targeting the chemokine receptor CXCR4 for cancer therapies. Biomark Res 13, 68. 40307933 10.1186/s40364-025-00778-y PMC12044942

